# Garetosmab in fibrodysplasia ossificans progressiva: a randomized, double-blind, placebo-controlled phase 2 trial

**DOI:** 10.1038/s41591-023-02561-8

**Published:** 2023-09-28

**Authors:** Maja Di Rocco, Eduardo Forleo-Neto, Robert J. Pignolo, Richard Keen, Philippe Orcel, Thomas Funck-Brentano, Christian Roux, Sami Kolta, Annalisa Madeo, Judith S. Bubbear, Jacek Tabarkiewicz, Małgorzata Szczepanek, Javier Bachiller-Corral, Angela M. Cheung, Kathryn M. Dahir, Esmée Botman, Pieter G. Raijmakers, Mona Al Mukaddam, Lianne Tile, Cynthia Portal-Celhay, Neena Sarkar, Peijie Hou, Bret J. Musser, Anita Boyapati, Kusha Mohammadi, Scott J. Mellis, Andrew J. Rankin, Aris N. Economides, Dinko Gonzalez Trotter, Gary A. Herman, Sarah J. O’Meara, Richard DelGizzi, David M. Weinreich, George D. Yancopoulos, E. Marelise W. Eekhoff, Frederick S. Kaplan

**Affiliations:** 1 Department of Pediatrics, Unit of Rare Diseases, IRCCS Istituto Giannina Gaslini, Genoa, Italy; 2 Regeneron Pharmaceuticals, Tarrytown, NY USA; 3 Department of Medicine, Mayo Clinic, Rochester, MN USA; 4 Centre for Metabolic Bone Disease Royal National Orthopaedic Hospital NHS Trust, London, UK; 5 Department of Rheumatology - DMU Locomotion, Assistance Publique - Hôpitaux de Paris, Paris, France; 6 INSERM Université Paris Cité, Paris, France; 7 Department of Rheumatology, Cochin Hospital, Assistance Publique - Hôpitaux de Paris, Paris, France; 8 Institute of Medical Sciences, Medical College of Rzeszów University, Rzeszów University, Rzeszów, Poland; 9 Department of Rheumatology, Hospital Universitario Ramón y Cajal, Madrid, Spain; 10 University Health Network, University of Toronto, Toronto, Ontario Canada; 11 Vanderbilt University Medical Center, Program for Metabolic Bone Disorders, Nashville, TN USA; 12 Department of Endocrinology and Metabolism, Amsterdam University Medical Centers (UMC), Vrije Universiteit, Amsterdam UMC Expert Center in Rare Bone Disease, Amsterdam Movement Sciences, Amsterdam, The Netherlands; 13 Department of Radiology and Nuclear Medicine, Amsterdam UMC, Vrije Universiteit, Amsterdam, The Netherlands; 14 Departments of Orthopaedics, Medicine and the Center for Research in FOP & Related Disorders, University of Pennsylvania Perelman School of Medicine, Philadelphia, PA USA

**Keywords:** Connective tissue diseases, Musculoskeletal abnormalities, Clinical trials, Drug development, Musculoskeletal system

## Abstract

Fibrodysplasia ossificans progressiva (FOP) is a rare disease characterized by heterotopic ossification (HO) in connective tissues and painful flare-ups. In the phase 2 LUMINA-1 trial, adult patients with FOP were randomized to garetosmab, an activin A-blocking antibody (*n* = 20) or placebo (*n* = 24) in period 1 (28 weeks), followed by an open-label period 2 (28 weeks; *n* = 43). The primary end points were safety and for period 1, the activity and size of HO lesions. All patients experienced at least one treatment-emergent adverse event during period 1, notably epistaxis, madarosis and skin abscesses. Five deaths (5 of 44; 11.4%) occurred in the open-label period and, while considered unlikely to be related, causality cannot be ruled out. The primary efficacy end point in period 1 (total lesion activity by PET–CT) was not met (*P* = 0.0741). As the development of new HO lesions was suppressed in period 1, the primary efficacy end point in period 2 was prospectively changed to the number of new HO lesions versus period 1. No placebo patients crossing over to garetosmab developed new HO lesions (0% in period 2 versus 40.9% in period 1; *P* = 0.0027). Further investigation of garetosmab in FOP is ongoing. ClinicalTrials.gov identifier NCT03188666.

## Abstract (web summary)

In the LUMINA-1 trial for fibrodysplasia ossificans progressiva, garetosmab, an activin A monoclonal antibody, did not lead to significant changes in heterotopic ossification lesion activity in pre-existing lesions in period 1. Garetosmab prevented the formation of new lesions in both periods 1 and 2.

## Main

FOP (MIM 135100) is an ultra-rare disorder^1^ with an estimated prevalence of 0.36–1.36 per million^2–5^. Common manifestations include congenital valgus deformities of the great toe, as well as HO and inflammatory flare-ups in connective tissues^6^. HO in FOP is cumulative and results in joint immobility, skeletal deformity, severe pain, disability and early mortality^6–9^. The estimated median age of survival for patients with FOP is 56 years^10^. Mortality is correlated with disease severity and the cumulative analog joint involvement scale (CAJIS) score^11,12^ and is primarily due to cardiorespiratory failure, pneumonia and complications of falls^10^.

FOP is caused by heterozygous missense mutations in *ACVR1*, the gene encoding activin A receptor type 1, a bone morphogenetic protein (BMP) type I receptor^13^. FOP-causing variants of *ACVR1* recognize activin A as an agonist, whereas wild-type ACVR1 does not (Supplementary Fig. 1)^14–17^. Garetosmab, a fully human monoclonal antibody (generated using VelocImmune technology)^18,19^, binds activin A and blocks its ability to activate FOP-mutant *ACVR1*. Garetosmab blocked the emergence of new HO lesions and stopped the growth or induced regression of pre-existing lesions in a genetically humanized murine FOP model^14,15^. We hypothesized that garetosmab, through blockade of activin A, may beneficially impact FOP progression in humans and designed LUMINA-1 to rigorously assess safety and efficacy as a potential disease-modifying therapy, as well as to clinically validate the role of activin A as a key driver of disease.

## Results

**Fig. 1:**
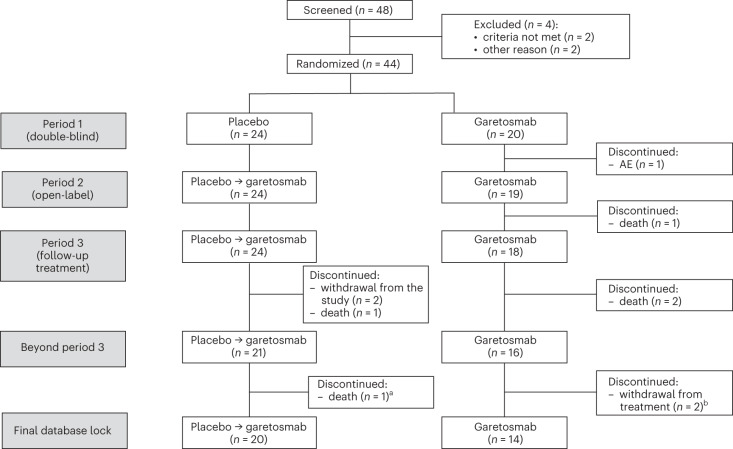
Trial profile: LUMINA-1 CONSORT flow diagram. A total of 48 patients were screened and 44 patients were randomized (20 patients to garetosmab and 24 patients to placebo). One patient from the garetosmab group discontinued from the study in period 1 due to a TEAE of pyrexia and four patients discontinued during period 2/period 3 due to potential risks associated with the COVID-19 pandemic (*n* = 1) and a lack of perceived benefit (*n* = 3). As of the final database lock (20 October 2021), five patients had died. Period 3 was the open-label extension portion of the trial that extended beyond 56 weeks. ^a^Patient died beyond period 3 (after week 76). ^b^These patients decided to withdraw from treatment due to lack of perceived benefit compared to the AE experienced. Both patients continued for safety follow-up.

LUMINA-1 enrolled 44 adult patients with FOP, all of whom had active HO at baseline (AHO population). Among these, 42 of 44 (95.5%) patients had the ‘classic’ FOP-causing variant of *ACVR1* (c.617 G > A, p.R206H; active heterotopic ossification classic (AHOC) population). Patients were randomized to intravenous (i.v.) garetosmab 10 mg kg^−1^ every 4 weeks (Q4W) (*n* = 20) or placebo (*n* = 24) in period 1 (Fig. 1 and Supplementary Fig. 2). In period 2, where all patients received open-label garetosmab, 42 of 44 (95.5%) patients were included in the intent-to-treat (ITT) population, with 40 of 44 (90.9%) patients included in the AHO coronavirus disease 2019 (COVID-19) modified ITT (mITT) population, defined as a result of constraints to the trial conduct during the COVID-19 pandemic. Dosing was suspended during the open-label extension portion of the trial (period 3 that extended beyond 56 weeks) due to a fatal adverse event (AE) that was initially and incorrectly believed to be bleeding-related. Following analysis of period 1 results, the hypothesis and end point analyses for period 2 were prospectively redefined in a protocol amendment. For the period 2 primary and key secondary end points, the analyses were conducted in the 22 patients who crossed over from placebo to garetosmab for a within-group comparison. Baseline demographics and disease characteristics were similar between treatment groups, including participation of both females and males (Table 1). The mean (s.d.) age was 27.6 (8.5) years. The mean CAJIS score at baseline was 15.7 (s.d. 6.6; range, 6–30; median (quartile 1, quartile 3), 15.0 (11, 19)). The study was initiated in February 2018; the primary data cutoff was 17 September 2019 (week 28); additional data cutoff dates for efficacy analyses were 11 August 2020 (week 56) and 30 October 2020 (week 76); and safety was reported until the last patient’s last visit on 16 September 2021.

**Table 1:** Baseline demographics and disease characteristics^a^

	Placebo (*n* = 24)	Garetosmab 10 mg kg^−1^ Q4W (*n* = 20)	Total (*N* = 44)
**Demographics**			
Age, years, mean (s.d.)	27.8 (8.5)	27.3 (8.7)	27.6 (8.5)
Ethnicity, *n* (%)			
Not Hispanic or Latino	24 (100)	20 (100)	44 (100)
Race, *n* (%)			
White	22 (91.7)	17 (85.0)	39 (88.6)
Black or African American	1 (4.2)	0	1 (2.3)
Asian	1 (4.2)	2 (10.0)	3 (6.8)
Other	0	1 (5.0)	1 (2.3)
Sex, *n* (%)			
Female	14 (58.3)	11 (55.0)	25 (56.8)
Weight, kg, mean (s.d.)	64.0 (23.4)	57.4 (10.1)	61.0 (18.7)
Region, *n* (%)			
North America	6 (25.0)	4 (20.0)	10 (22.7)
Europe	18 (75.0)	16 (80.0)	34 (77.3)
**Clinical characteristics**			
Age at FOP diagnosis, years, mean (s.d.)	8.1 (7.5)	9.1 (5.5)	8.5 (6.6)
Duration of FOP disease, years, mean (s.d.)	19.9 (10.0)	18.3 (11.0)	19.2 (10.4)
FOP genetic mutation, *n* (%)			
R206H (classic)	22 (91.7)	20 (100)	42 (95.5)
Other	2 (8.3)	0	2 (4.5)
FEV_1_, l, mean (s.d.)^b^	1.9 (0.8)	1.8 (0.6)	1.9 (0.7)
Percent predicted FEV_1_, %, mean (s.d.)^b^	53.2 (19.1)	53.0 (15.8)	53.1 (17.4)
FVC, l, mean (s.d.)^b^	2.1 (0.9)	2.0 (0.7)	2.1 (0.8)
P1NP, μg l^−1^, mean (s.d.)	138.1 (100.4)	182.9 (168.5)	158.5 (135.8)
BSAP, U l^−1^, mean (s.d.)	32.8 (14.8)	32.4 (15.9)	32.7 (15.1)
tAP, IU l^−1^, mean (s.d.)	76.8 (25.0)	72.9 (29.0)	75.0 (26.7)
**Patient- and physician-reported outcomes**			
NRS, mean (s.d.)^c^			
Average daily pain	1.96 (2.175)	2.04 (2.071)	1.99 (2.104)
Average daily pain over 7 d	2.17 (2.246)	2.01 (1.805)	2.10 (2.041)
Patients with flare-ups in previous 12 months, *n* (%)^d^	20 (83.3)	19 (95.0)	39 (88.6)
Total joint function score (CAJIS), mean (s.d.)^e^	15.7 (6.2)	15.8 (7.3)	15.7 (6.6)
EQ-5D-3L total score, mean (s.d.)	9.4 (1.5)	9.2 (2.1)	9.3 (1.8)
FOP I-ADL, mean (s.d.)	69.3 (26.3)	77.9 (35.9)	73.2 (30.9)
**Imaging characteristics**			
Presence of active HO at baseline, *n* (%)^f^	24 (100)	20 (100)	44 (100)
Number of active HO lesions by ^18^F-NaF PET, *n* (%)			
1	0	0	0
2	3 (12.5)	1 (5.0)	4 (9.1)
3	1 (4.2)	1 (5.0)	2 (4.5)
4	2 (8.3)	2 (10.0)	4 (9.1)
5	1 (4.2)	2 (10.0)	3 (6.8)
6	4 (16.7)	1 (5.0)	5 (11.4)
7	13 (54.2)	13 (65.0)	26 (59.1)
TLA by ^18^F-NaF PET, mean (s.d.)	473.4 (348.4)	418.2 (372.8)	448.3 (356.5)
Number of HO lesions by CT, *n* (%)			
1	0	0	0
2	3 (12.5)	1 (5.0)	4 (9.1)
3	2 (8.3)	2 (10.0)	4 (9.1)
4	1 (4.2)	1 (5.0)	2 (4.5)
5	1 (4.2)	2 (10.0)	3 (6.8)
6	6 (25.0)	1 (5.0)	7 (15.9)
7	11 (45.8)	13 (65.0)	24 (54.5)
Total volume of HO lesions by CT, cm^3^, mean (s.d.)	235.8 (253.3)	251.4 (327.9)	242.9 (286.2)

^a^AHO.

^b^Spirometry, *n* = 22 (placebo), *n* = 19 (garetosmab 10 mg kg^−1^ Q4W) and *n* = 41 (total).

^c^NRS, *n* = 23 (placebo), *n* = 18 (garetosmab 10 mg kg^−1^ Q4W) and *n* = 41 (total).

^d^Patient e-diary.

^e^Physician assessment.

^f^Defined as ≥1 lesion with an SUV_max_ that is ≥3 times the SUV_mean_ for the supra-acetabular region of interest.

^18^F-NaF PET, fluorine-18-labeled sodium fluoride positron emission tomography; AHO, baseline active heterotropic ossification analysis set; BSAP, bone-specific alkaline phosphatase, reference range in adults 11.6–43.4 U l^−1^; EQ-5D-3L, EuroQol five dimensions questionnaire with a three-level scale; FEV_1_, forced expiratory volume in 1 s; FVC, forced vital capacity; I-ADL, instrumental activities of daily living; P1NP, procollagen type 1 N-terminal propeptide, reference range in adults 13.3–97 μg l^−1^; SUV_max_, maximal standardized uptake value; SUV_mean_, mean standardized uptake value; tAP, total alkaline phosphatase, reference range in adults 37–116 IU l^−1^.

### Safety overview (periods 1–2 and open-label extension)

In period 1, all 44 patients experienced at least one treatment-emergent AE (TEAE; Table 2a). Garetosmab was associated with more AEs than placebo; particularly epistaxis, madarosis (loss of eyebrows/eyelashes) and skin/soft-tissue infections (see below).

**Table 2:** a, Primary safety end point of incidence and severity of TEAEs during the double-blind period of the study (period 1)^a^. b, TEAEs that occurred in ≥10% in period 2 and the open-label extension period until the end of the study^a,b^

a, Period 1 results		
Patients, *n* (%)	Placebo (*n* = 24)	Garetosmab 10 mg kg^−1^ Q4W (*n* = 20)
≥1 TEAE	24 (100)	20 (100)
≥1 SAE	2 (8.3)	4 (20.0)
≥1 severe TEAE	3 (12.5)	3 (15.0)
≥1 drug-related TEAE	13 (54.2)	13 (65.0)
≥1 TEAE resulting in discontinuation from study	0	1 (5.0)
≥1 TEAE resulting in death	0	0
≥1 TEAE of AESI^c^	0	1 (5.0)
TEAEs occurring in ≥4 patients in any treatment group^d^		
Headache	7 (29.2)	10 (50.0)
Epistaxis	4 (16.7)	10 (50.0)
Acne	3 (12.5)	6 (30.0)
Pain in extremity	9 (37.5)	5 (25.0)
Arthralgia	9 (37.5)	7 (35.0)
Diarrhea	6 (25.0)	5 (25.0)
Madarosis	0	6 (30.0)
Back pain	1 (4.2)	4 (20.0)
Neck pain	3 (12.5)	4 (20.0)
Toothache	0	4 (20.0)
Rhinitis	0	4 (20.0)
Dizziness	2 (8.3)	4 (20.0)
Nasopharyngitis	5 (20.8)	3 (15.0)
Rash	4 (16.7)	2 (10.0)

b, Period 2 results	
Primary system organ class preferred term	Total (*N* = 43)
TEAEs, *n*	865
Patients with ≥1 TEAE, *n* (%)	43 (100)
Infections and infestations, *n* (%)	37 (86.0)
Nasopharyngitis	14 (32.6)
Limb abscess	6 (14.0)
Rhinitis	5 (11.6)
Anal abscess	4 (9.3)
Folliculitis	4 (9.3)
Furuncle	4 (9.3)
Gastroenteritis	4 (9.3)
Paronychia	4 (9.3)
Abdominal abscess	2 (4.7)
Groin abscess	2 (4.7)
Hordeolum	2 (4.7)
Subcutaneous abscess	2 (4.7)
Vulval abscess	2 (4.7)
Skin and subcutaneous tissue disorders, *n* (%)	35 (81.4)
Madarosis	20 (46.5)
Acne	14 (32.6)
Alopecia	8 (18.6)
Rash	6 (14.0)
Hirsutism	4 (9.3)
Erythema	3 (7.0)
Hypertrichosis	3 (7.0)
Decubitus ulcer	2 (4.7)
Musculoskeletal and connective tissue disorders, *n* (%)	25 (58.1)
Arthralgia	15 (34.9)
Pain in extremity	12 (27.9)
Back pain	10 (23.3)
Musculoskeletal pain	5 (11.6)
Neck pain	5 (11.6)
Spinal pain	5 (11.6)
Myalgia	3 (7.0)
Muscular weakness	2 (4.7)
Gastrointestinal disorders, *n* (%)	24 (55.8)
Diarrhea	7 (16.3)
Nausea	7 (16.3)
Vomiting	4 (9.3)
Aphthous ulcer	3 (7.0)
Mouth ulceration	3 (7.0)
Injury, poisoning and procedural complications, *n* (%)	22 (51.2)
Post-traumatic pain	6 (14.0)
Contusion	5 (11.6)
Joint injury	4 (9.3)
Skin laceration	3 (7.0)
Respiratory, thoracic and mediastinal disorders, *n* (%)	22 (51.2)
Epistaxis	15 (34.9)
Cough	5 (11.6)
Oropharyngeal pain	3 (7.0)
Rhinorrhea	3 (7.0)
General disorders and administration site conditions, n (%)	19 (44.2)
Pyrexia	11 (25.6)
Pain	3 (7.0)
Swelling	2 (4.7)
Nervous system disorders, *n* (%)	19 (44.2)
Headache	13 (30.2)
Dizziness	4 (9.3)
Reproductive system and breast disorders, *n* (%)	9 (20.9)
Ovarian cyst	3 (7.0)
Ear and labyrinth disorders, *n* (%)	6 (14.0)
Hypoacusis	3 (7.0)
Ear discomfort	2 (4.7)

^a^Safety analysis set.

^b^A patient with multiple TEAEs is counted once for the same preferred term or system organ class. This table is sorted by descending order of frequency of system organ class and preferred term for the treatment group.

^c^AESIs included epididymitis, orchitis, hydrocele, scrotum pain, scrotum swelling, moderate to severe episodes of non-traumatic bleeding, moderate epistaxis (≥30 min or requiring professional medical intervention) and severe epistaxis (based on the definition of a severe TEAE).

^d^Preferred term.

AESI, AE of special interest.

**Extended Data Fig. 1:**
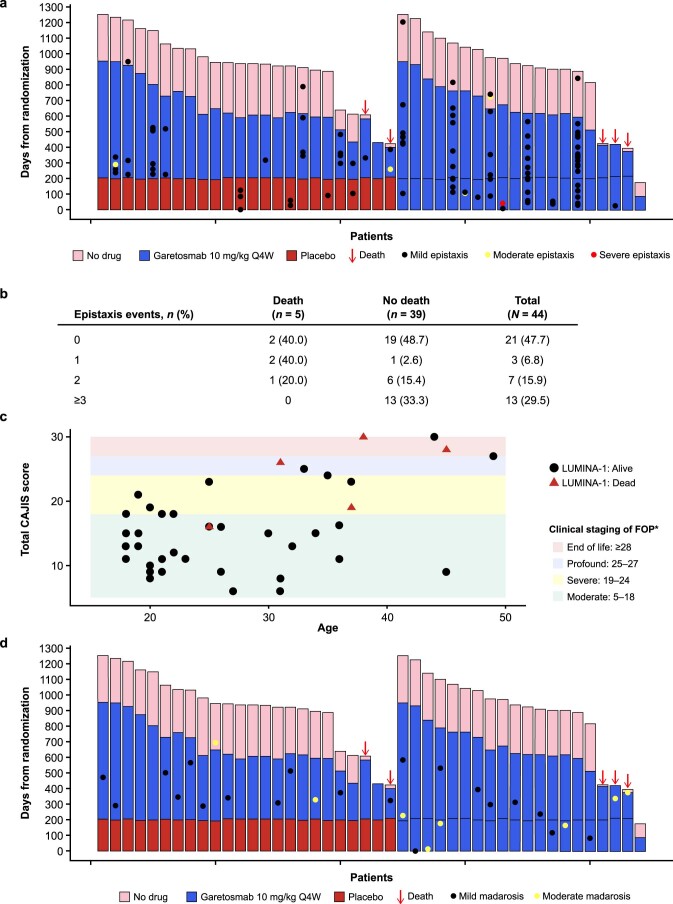
Lack of association of epistaxis events and deaths in the LUMINA-1 study, number of epistaxis TEAEs in patients with or without fatal SAEs, relationship between patient baseline age and disease severity as measured by the clinical staging of FOP^a^, and madarosis events in the LUMINA-1 study. **a**, In red are patients who received placebo during the 28-week period 1, and in blue are patients receiving garetosmab. Dots represent epistaxis events. Black dots are mild epistaxis events, yellow dots are moderate events (defined as any episode lasting longer than 30 mins or requiring medical intervention) and red dots are severe events. Red arrows indicate the patient experienced a fatal SAE. **b**, The table shows the number of epistaxis TEAEs in patients with or without fatal SAEs. No drug refers to the period after dosing was discontinued following clinical hold. **c**, Red triangles indicate patients with fatal SAEs. **d**, In red are patients who received placebo during the 28-week period 1, and in blue are patients who received garetosmab. Dots represent madarosis events: black dots are mild madarosis events and yellow dots are moderate events. Red arrows indicate the patient experienced a fatal SAE. ^a^CAJIS is an assessment of mobility limitation at 15 anatomic locations; scores are tabulated for each site as normal unaffected (0), affected (1), or completely functionally ankylosed (2). The total score ranges from 0 to 30. On the basis of FOP features (flare-up activity, body regions affected, thoracic insufficiency syndrome, other complications) and its consequences (impairments in activities of daily living and ambulation, increasing CAJIS), five clinical stages of disease severity have been defined^12^. In early-stage FOP the total CAJIS score is usually ≤4, in the moderate stage 5–18, in the severe stage 19–24, in the profound stage ≥24, and in the end-of-life stage ≥28. CAJIS, Cumulative Analog Joint Involvement Scale; FOP, fibrodysplasia ossificans progressiva; SAE, severe adverse event; TEAE, treatment-emergent adverse event.

Five deaths occurred during the open-label periods (5 of 44; 11.4%); case summaries can be found in Extended Data Table 1. The deaths were reported by investigators as unrelated to garetosmab and there was no clear pattern to link with the treatment or to the mechanism of action, although a causal relation cannot be excluded. The causes of death were head and brain trauma due to a fall in the setting of severe motor disability; hemorrhagic stroke in the setting of poorly controlled hypertension; fatal intestinal obstruction in the setting of a previous episode of intestinal obstruction; traumatic spleen rupture and cardiac arrest due to a fall; and sudden cardiac death in a patient with lung granulomatous inflammation most likely attributable to chronic pulmonary aspiration. There was no relationship between individuals who died and the frequency or severity of epistaxis (Extended Data Fig. 1a,b). Of the five deaths, three occurred in patients with a CAJIS score ≥24 and either profound or end-of-life disease severity as measured by the clinical staging of FOP developed by Pignolo and Kaplan (Extended Data Fig. 1c)^12^. Among the fatal events in study participants with lower CAJIS scores, one occurred in a 26–30-year-old patient (age ranges rather than exact age are provided to protect the identity of individual patients) with a CAJIS score of 16 (moderate disease staging) who died from a fall down a flight of stairs resulting in severe head and brain trauma. This individual had substantial rigidity and walking disability at baseline. Another was a 36–40-year-old patient with a CAJIS score of 19 (severe disease staging) who died from apparent sudden cardiac arrest with extensive granulomatous formation in the lungs consistent with chronic aspiration of foreign material.

Regarding other AEs, most were considered by investigators to be mild to moderate in severity. In period 1, notable imbalances in AEs with garetosmab compared to placebo included epistaxis (50.0% versus 16.7%), madarosis (30.0% versus 0%) and a composite of skin and soft-tissue infections that included acne (60% versus 12.5%; Table 2a and Supplementary Tables 1 and 2). Nine serious AEs (SAEs) in period 1 occurred in six (13.6%) patients; of these, four (20.0%) received garetosmab and two (8.3%) received placebo (Supplementary Table 3). SAEs in the garetosmab group included three (15.0%) patients with infections and infestations, one (5.0%) with epistaxis and one (5.0%) with intestinal obstruction. The SAE of epistaxis resulted in hospitalization for nasal packing and was assessed as related to garetosmab by the investigator. This patient required no transfusions, had no drop in hemoglobin, fully recovered and continued in the study without recurrence. This epistaxis SAE led to a protocol amendment to include additional exclusion criteria, as well as baseline (if appropriate samples were available) and post-treatment laboratory measures of coagulation parameters and platelet effector function to exclude patients who may have had existing propensity for bleeding and to mitigate the potential risk for epistaxis.

**Extended Data Fig. 2:**
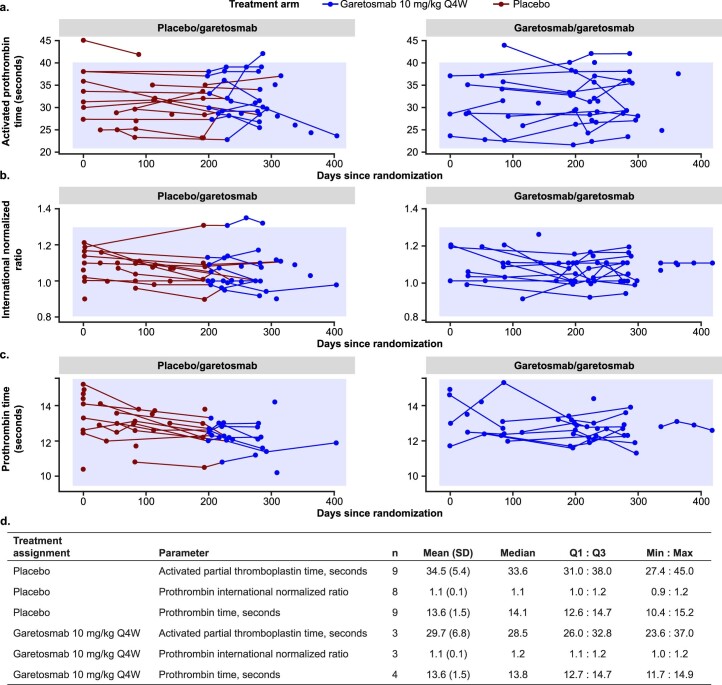
Coagulation and platelet functional assays at baseline and post-treatment in LUMINA-1 patients. Coagulation tests and platelet functional assays including **a**, activated prothrombin time **b**, prothrombin international normalized ratio and **c**, prothrombin time were collected after protocol amendment #3 at local laboratories to explore the mechanism of epistaxis and garetosmab mechanism of action. For patients already enrolled in the study, the blood samples for these assessments were collected at their next visit unless the assessment was performed in the last year. The blue box indicates the normal range across the local labs for the individual sites. **d**, The table shows the number of measurements and summary statistics for the coagulation tests and platelet functional assays at baseline. Q4W, every 4 weeks; SD, standard deviation.

Coagulation tests and platelet functional assays, including prothrombin time, activated prothrombin time and prothrombin international normalized ratio, measured in a subset of patients at baseline and post-treatment, were in the normal range at baseline; fluctuations observed in the placebo and garetosmab groups remained within the normal ranges (Extended Data Fig. 2). In period 1, 13 (65.0%) patients in the garetosmab group experienced a bleeding event compared to nine (37.5%) patients in the placebo group (Supplementary Table 4). The only bleeding event reported in more than two patients in the garetosmab group was epistaxis. Other bleeding events were balanced between garetosmab and placebo recipients and were non-serious. No patients discontinued therapy due to epistaxis. One patient with a medical history of restrictive lung disease, pulmonary congestion and mucus plugging discontinued the study due to a TEAE of mild pyrexia that followed recurrent episodes of pneumonia and hemoptysis.

In period 2 and the subsequent open-label extension period, all patients received garetosmab and reported at least one AE. The most frequently reported AEs were consistent with those reported in period 1, consisting of skin and soft-tissue infections (81.4%; acne (32.6%), madarosis (46.5%)) and epistaxis (34.9%) and were mostly mild to moderate in severity (Table 2b and Extended Data Fig. 1a,d). Epistaxis was reported in 15 (34.9%) patients. Most events were mild and no patients discontinued treatment. Twenty SAEs in 13 (30.2%) patients were reported, including infections and infestations (16.3%) and gastrointestinal disorders (4.7%; Supplementary Table 5). Five (11.6%) patients experienced six SAEs of abscess requiring a hospital admission or emergency room visit for incision and drainage. These events resolved and the patients continued garetosmab after a temporary interruption; five of these six SAEs were deemed related to garetosmab by the investigator.

To determine whether epistaxis events were related to changes in BMP/transforming growth factor-β family members (other than activin A) known to regulate angiogenesis and vascular endothelium homeostasis, levels of BMP9 were measured^20,21^. Minor fluctuations in the level of BMP9 were observed in both the garetosmab and placebo groups, however, these fluctuations did not correlate with episodes of epistaxis (Supplementary Fig. 3).

The percentage of patients with infusion reactions was balanced between garetosmab (5 of 20; 25%) and placebo (6 of 24; 25%). Some reactions required a temporary infusion interruption or antihistamine (loratadine) pre-medication (*n* = 2), but all infusions were completed. No patient discontinued treatment due to infusion reactions and none of the infusion reactions were associated with signs or symptoms of anaphylaxis or the development of antidrug antibodies.

### Period 1 efficacy results

**Fig. 2:**
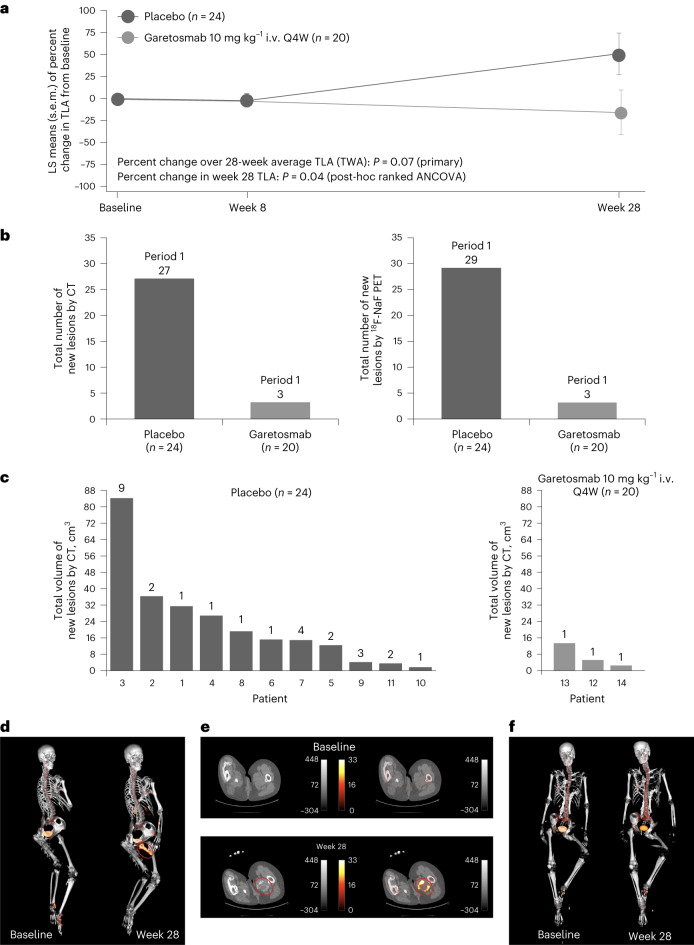
Effect of garetosmab on the change from baseline in TLA compared to placebo, the total number of new HO lesions assessed by quantitative imaging and the total volume of new HO lesions by CT per patient in period 1 and representative images. **a**, TWA of the percent change over 28 weeks in TLA (TWA (±s.e.m.) 16.6 (9.1) versus −8.1 (0.9); *P* = 0.07 (primary)) as assessed by ^18^F-NaF PET (AHO) was analyzed through an ANCOVA model. **b**, Total number of new lesions in all patients per group (combined) by CT and ^18^F-NaF PET during period 1 relative to baseline (AHO). **c**, Total volume of new lesions per patient as assessed by CT in period 1 (AHO); number of new lesions per patient (bold text on top of each column bar) as assessed by CT in period 1 (AHO). **d**, Surface-rendered baseline (left) and week 28 (right) PET–CT fusion images of a placebo-treated patient with a prominent femoral bridge new HO lesion detectable at week 28 by PET–CT (inside red oval). The PET signal in the arm is due to the site of tracer injection. **e**, Transaxial CT (left) and fused PET–CT (right) images of baseline (top) and week 28 (bottom) of the same patient from Panel **d**, displaying detail of the prominent new HO lesion (inside red oval) with a blend of high- and low-density regions evident on CT and corresponding high uptake by PET, indicating a high rate of mineralization compared to normotopic and mature HO present on the contralateral leg (the grayscale color bar indicates Hounsfield units (HU) and the hot iron color bar shows PET SUV units). **f**, A garetosmab-treated patient showing no HO-related PET–CT changes between baseline and week 28 scans. For both patients, the PET signal in the area of the pubis is normal urinary bladder uptake, signal near the spine and below the ribs is normal kidney uptake and signal in the feet correspond to non-HO bone degenerative disease. Apparent bone projection from the left femur at week 28 is an image artifact (**e**).

Forty-three (98%) patients completed period 1 and all prespecified primary and secondary end points under type I error control for period 1 are reported in Table 3a. All 44 randomized patients were included in the AHO population and 42 were included in the AHOC population. The prespecified primary efficacy end points assessed the impact of garetosmab on the activity (positron emission tomography (PET)) and volume (computed tomography (CT)) of HO lesions pre-existing at baseline, as well as those newly appearing. The primary end point of time-weighted percent change from baseline in total lesion activity (TLA) by PET–CT was not met in period 1. The percent change from baseline in TLA, compared to placebo, was −24.6% in the AHO population (95% confidence interval (CI) −51.8, 2.5; *P* = 0.07; Fig. 2a and Table 3a). As the first primary efficacy end point was nonsignificant, the hierarchical analysis was stopped and so *P* values are not reported for the other end points listed in Table 3a. In period 1 (AHO population), for the volumetric change in HO volume by CT from baseline to week 28, the least squares (LS) mean difference between garetosmab and placebo was −24.9% (95% CI −80.8, 30.9). Additionally, the average change from baseline in daily pain was compared between the garetosmab and placebo arms in the AHO population (LS mean difference: −0.34 (95% CI −0.96, 0.27)). Similar results were observed for analogous end points in the AHOC population. Other secondary end points (not predefined in the hierarchy) for period 1 are reported in Supplementary Table 6a.

**Table 3:** Summary of prespecified primary and key secondary end points for (a) period 1 and (b) period 2

a, Period 1 results							
End point	Order of hierarchy	Population	Model^a^	Garetosmab LS mean (s.e.m.)	Placebo LS mean (s.e.m.)	LS mean difference (95% CI)	*P* value^b^
Primary: TWA of percent change from baseline in TLA by ^18^F-NaF PET over 28 weeks	1	AHO	ANCOVA	−8.1 (9.93)	16.6 (9.11)	−24.6 (−51.8, 2.5)	0.0741
Primary: percent change from baseline in total volume of HO lesions by CT at week 28	2	AHO	MMRM	7.1 (20.43)	32.0 (18.66)	−24.9 (−80.8, 30.9)	-
Primary: TWA of percent change from baseline in TLA by ^18^F-NaF PET over 28 weeks	3	AHOC	ANCOVA	−8.0 (10.14)	17.6 (9.73)	−25.6 (−53.9, 2.8)	-
Primary: percent change from baseline in total volume of HO lesions by CT at week 28	4	AHOC	MMRM	7.0 (20.87)	34.9 (19.90)	−27.8 (−86.1, 30.5)	-
Key secondary: TWA of change from baseline in daily average pain over 28 weeks	5	AHO	ANCOVA	−0.51 (0.231)	−0.17 (0.21)	−0.34 (−0.96, 0.27)	-
	6	AHOC	ANCOVA	−0.48 (0.237)	−0.12 (0.22)	−0.36 (−1.01, 0.29)	-

b, Period 2 results (COVID-19 mITT analysis set)								
End point	Order of hierarchy	Arm^c^	*n*	Model	Period 1	Period 2	Comparison of period 1 versus period 2	*P* value^d^
Number of new HO lesions as assessed by CT at week 56 relative to week 28 scan	1	Placebo/garetosmab	22	Descriptive + Wilcoxon	Observed rate = 1 Total no. of lesions = 22	Observed rate = 0 Total no. of lesions = 0	Observed rate reduction = 100%	0.0039
	–	Garetosmab/garetosmab	18	Descriptive	Observed rate = 0.11 Total no. of lesions = 2	Observed rate = 0 Total no. of lesions = 0		
Total volume of new HO lesions as assessed by CT at week 56 relative to week 28 scan	2	Placebo/garetosmab	22	MMRM + Wilcoxon	LS mean 9.29 cm^3^	LS mean 0.05 cm^3^	LS mean difference, −9.24 (95% CI −17.96, −0.52)	0.0039
	–	Garetosmab/garetosmab	18	Descriptive	Mean 1 cm^3^	Mean 0 cm^3^		
Number of new HO lesions as assessed by ^18^F-NaF PET at week 56 relative to week 28 scan	3	Placebo/garetosmab	22	GEE + Wilcoxon	Adjusted rate 0.93 (95% CI 0.54, 1.62) Total no. of lesions = 23	Adjusted rate 0.04 (95% CI 0.01, 0.31) Total no. of lesions = 1	Adjusted rate ratio, 0.05 (95% CI 0.01, 0.33) Rate reduction = 95%	0.0039
	–	Garetosmab/garetosmab	18	Descriptive	Observed rate = 0.06 Total no. of lesions = 1	Observed rate = 0 Total no. of lesions = 1		
TLA by ^18^F-NaF PET in new lesions at week 56 relative to week 28 scan	4	Placebo/garetosmab	22	MMRM + Wilcoxon	LS mean 204.45	LS mean 13.20	LS mean difference, −191.25 (95% CI −390.80, 8.29)	0.0273
	–	Garetosmab/garetosmab	18	Descriptive	Mean 4.7	Mean 0		
Percent of patients with new HO lesions as assessed by CT at week 56 relative to week 28	5	Placebo/garetosmab	22	Descriptive + McNemar	40.9 (9 of 22)	0 (0 of 22)	Observed relative risk reduction = 100%	0.0027
	–	Garetosmab/garetosmab	18	Descriptive	11.1 (2 of 18)	0 (0 of 18)		
Percent of patients with new HO lesions as assessed by PET at week 56 relative to week 28	6	Placebo/garetosmab	22	GEE + McNemar	40.9 (9 of 22)	4.5 (1 of 22)	Adjusted relative risk reduction = 89% Adjusted odds ratio, 0.07 (95% CI 0.01, 0.48)	0.0047
	–	Garetosmab/garetosmab	18	Descriptive	5.6 (1 of 18)	0 (0 of 18)		

^a^For ANCOVA and MMRM, LS mean (s.e.m.) and LS mean differences (95% CI) are shown.

^b^Testing of the primary and key secondary efficacy end points followed a hierarchical testing sequence only if statistical significance was established for all primary end points (*P* value < 0.05). The first primary end point was not statistically significant therefore only effect sizes and 95% CI were given for the other end points.

^c^The key statistical comparisons were based on within-group comparisons for the patients who were randomized to placebo in the double-blind period and crossed over to the garetosmab group in the open-label period (period 2). Those who received garetosmab for both periods are included in this table; however, they were not in the period 2 hierarchy.

^d^To control for the type I error rate at 0.10 for the primary and key secondary null hypotheses in period 2, a hierarchical testing procedure was applied at a two-sided 10% significant level (*P* values < 0.1 are considered statistically significant).

^18^F-NaF, fluorine-18-labeled sodium fluoride; AHO, patients with at least one active HO lesion at baseline; AHOC, patients with at least one active HO lesion at baseline and with classic *ACVR1*^R206H^ mutation; ANCOVA, analysis of covariance; COVID-19 mITT, coronavirus disease 2019 modified intention to treat analysis set; GEE, general estimating equation; LS, least squares; MMRM, mixed model with repeated measures.

**Extended Data Fig. 3:**
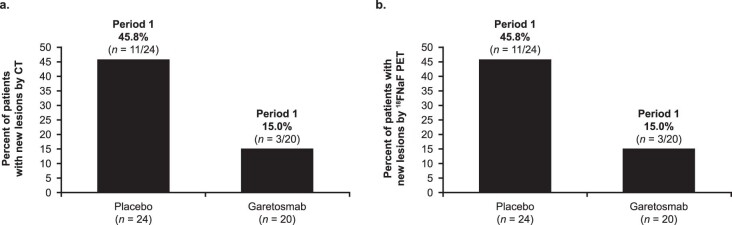
CT and ^18^F-NaF PET: Percent of patients with new lesions from baseline to week 28. **a**, Percent of patients with new lesions by CT during period 1 relative to baseline (AHO). **b**, Percent of patients with new lesions by ^18^F-NaF PET during period 1 relative to baseline (AHO). ^18^F-NaF, fluorine-18-labeled sodium fluoride; AHO, active HO analysis set; CT, computed tomography; HO, heterotopic ossification; PET, positron emission tomography.

**Extended Data Fig. 4:**
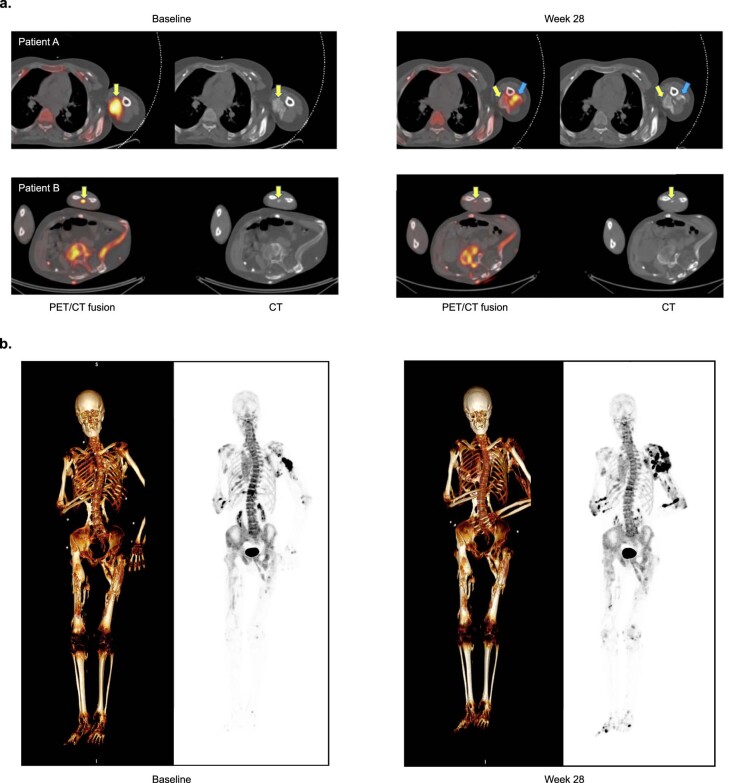
Representative CT and PET images of patients treated with garetosmab and placebo. **a**, Baseline and week 28 PET/CT fusion and CT scans for Patient A (placebo group) and Patient B (garetosmab group). At baseline, Patient A showed a large HO lesion next to the humerus (shown by the yellow arrow), the PET signal of which decreased from an SUV_mean_ of 33.46 to 12.25 and the volume of which by CT increased from 17.74 cm^3^ to 27.8 cm^3^, while a new lesion (shown by the blue arrow) with an SUV_mean_ of 23.49 and a CT volume of 10.1 cm^3^ was detectable at the 28-week scan. Patient B showed a small HO lesion, the PET signal of which decreased from an SUV_mean_ of 13.57 to 6.18 and the CT volume of which increased slightly from 1.72 cm^3^ to 2.12 cm^3^. **b**, Baseline (left panel) and week 28 (right panel) surface-rendered CT and maximum-intensity projection PET images of a placebo patient showing several prominent new lesions detectable by CT (bone bridges forming in the biceps and deltoid muscle regions indicated by green arrows) and PET (high-intensity uptake in the deltoid muscle region indicated by orange arrows). New foci of PET uptake in the feet were deemed non-HO-related by the independent blinded readers. CT, computed tomography; HO, heterotopic ossification; PET, positron emission tomography; SUV_mean_, mean standardized uptake value.

Post-hoc analyses of newly appearing (‘new’) lesions showed a ~97% relative reduction in new lesion activity, as well as a ~90% relative reduction in new HO lesion volume, when comparing the garetosmab and placebo arms (Fig. 2b–f and Supplementary Tables 7 and 8). Pre-existing lesions did not expand in volume in either the placebo or garetosmab groups. Additionally, reductions in the number of new HO lesions per patient as assessed by both PET (mean of 0.15 new lesions/patient for garetosmab versus 1.19 for placebo; rate ratio, 0.13; post-hoc analysis) and CT (0.15 versus 1.13, respectively; rate ratio, 0.13; post-hoc analysis; Supplementary Table 7) were observed. The total number of new HO lesions in period 1 for placebo was 27 by CT and 29 by PET compared to three by CT and three by PET for garetosmab (Fig. 2b). The percentage of patients who developed new lesions in period 1 was lower with garetosmab (15% by CT; 15% by PET) than with placebo (45.8% by both CT and PET; relative risk = 0.33 for PET and CT; Extended Data Fig. 3 and Supplementary Table 7). Representative images of patients treated with garetosmab and placebo are shown in Fig. 2d–f and Extended Data Fig. 4.

**Extended Data Fig. 5:**
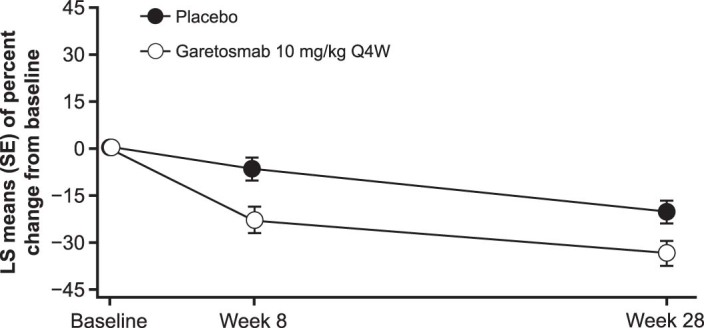
Percent change from baseline in mean ^18^F-NaF SUV_max_ per patient by PET over time for pre-existing lesions (AHO). LS means and SEs were estimated from ANCOVA. This model included treatment group and gender, as well as the continuous covariates of baseline mean SUV_max_. ^18^F-NaF, fluorine-18-labeled sodium fluoride; AHO, active heterotopic ossification analysis set; ANCOVA, analysis of covariance; BL, baseline; LS, least squares; PET, positron emission tomography; Q4W, every 4 weeks; SE, standard error; SUV_max_, maximal standardized uptake value.

**Extended Data Fig. 7:**
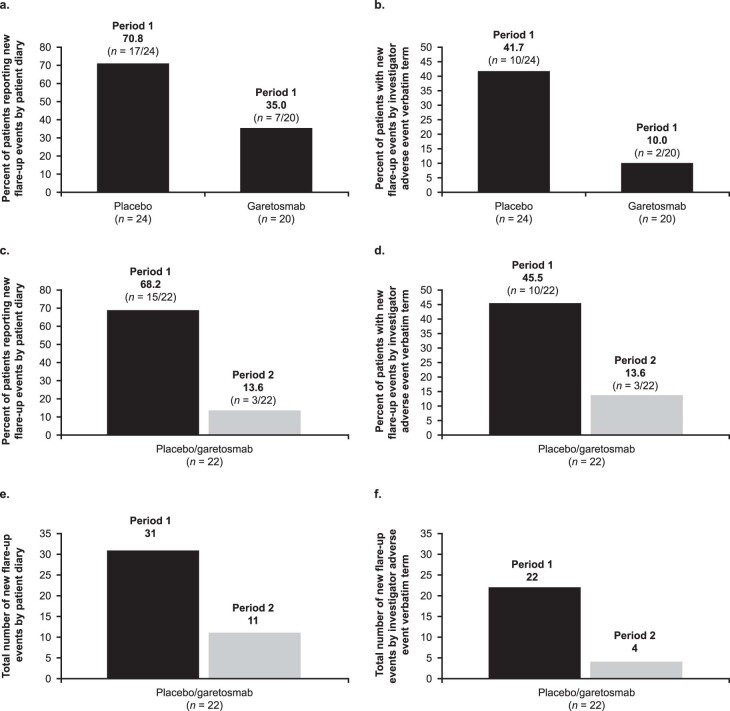
Effect of garetosmab on flare-up events. Percent of patients with new flare-up events as reported by **a**, patient diary and **b**, investigator AE verbatim term in period 1 (active HO analysis set). Panels C–E describe patients who crossed over from placebo in period 1 to garetosmab in period 2. Percent of patients with new flare-up events as reported by **c**, patient diary and **d**, investigator AE verbatim term for patients originally assigned to placebo (mITT analysis set). Total number of new flare-up events as reported by **e**, patient diary and **f**, investigator AE verbatim term for patients originally assigned to placebo (mITT analysis set). A new flare-up was defined as a flare-up event starting in the corresponding period. Investigator-defined flare-ups were defined as TEAEs with the verbatim term containing ‘flare’. Nominal *P* values are not shown for post-hoc analyses, that is, panels A–D. AE, adverse event; HO, heterotopic ossification; mITT, modified intent-to-treat; TEAE, treatment-emergent adverse event.

Examination of garetosmab’s impact on pre-existing lesions (PET *n* = 257 and CT *n* = 253) additionally showed that garetosmab reduced the maximal standardized uptake value of ^18^F-NaF in pre-existing lesions by 22.6% (week 8) and 33.2% (week 28), versus 6.4% and 20.2%, respectively, with placebo (post-hoc analyses; Extended Data Fig. 5)^15,22^. Garetosmab had no effect on the time-weighted average (TWA) percent change from baseline versus placebo for TLA and the total volume of pre-existing HO lesions at week 28 (post-hoc analyses, TLA: LS mean difference, 0.5; 95% CI −14.3, 15.2; HO volume: LS mean difference, −4.8; 95% CI −23.6, 14.1; Extended Data Fig. 7).

Because garetosmab was observed to block the formation of new HO lesions, its effects on normal skeletal bone were also examined using PET. Assessment of percent change from baseline of the mean (s.e.m.) standardized uptake value (SUV) of selected normotopic bones (a prespecified exploratory end point) showed no differences between treatments at week 8 (garetosmab: 42.0% (9.9); placebo: 20.3% (9.2); LS mean difference: 21.7 (95% CI −5.7, 49.1)) and week 28 (garetosmab: 24.6% (8.4); placebo: 28.9% (7.7); LS mean difference −4.2 (95% CI −27.3, 18.8)). Additionally, an initial exploratory analysis of C-terminal telopeptide 1 concentrations, a biomarker of bone resorption, showed that concentrations were within the normal reference range for males (0.016–0.704 ng ml^−1^) and females (pre-menopausal 0.025–0.573 ng ml^−1^; post-menopausal 0.104–1.008 ng ml^−1^) at all time points during garetosmab treatment^23,24^.

Soft-tissue inflammatory flare-ups events were nominally significantly reduced as reported by patients on garetosmab compared to those on placebo based on daily diary entries (35.0% versus 70.8%, respectively; RR = 0.49; prespecified exploratory end point). Investigator’s reports of AEs were explored for flare-ups and reductions were also observed in the garetosmab group (10.0% versus 41.7%, respectively; RR = 0.24; post-hoc analysis; Supplementary Table 9 and Extended Data Fig. 7a,b).

As period 1 prespecified secondary end points showed that existing lesions did not expand and that garetosmab profoundly reduced new HO lesion activity and growth, the study hypothesis for period 2 was prospectively redefined in protocol amendment no. 6 as follows: ‘garetosmab prevents the formation of new HO lesions’ and in an amended statistical analysis plan. Specifically, primary and key secondary efficacy end point analyses at week 56 focused on patients who crossed over from placebo to garetosmab and examined the number (primary), volume and activity (secondary) of new HO lesions that developed during period 2 compared to the number, volume and activity of new HO lesions that developed during period 1.

### Period 2 efficacy results

**Fig. 3:**
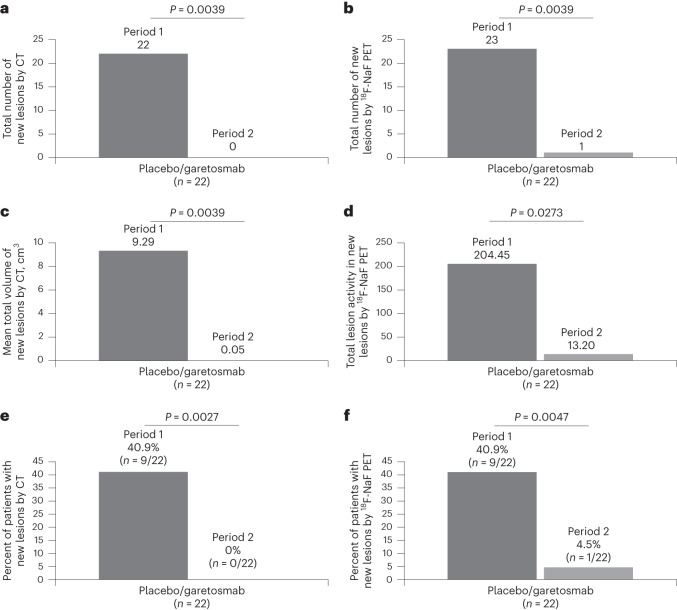
Effect of garetosmab in period 2 relative to period 1 on the total number of new HO lesions, the mean total volume of new HO lesions, the TLA in new HO lesions and the percentages of patients with new lesions assessed by quantitative imaging in those originally randomized to placebo. **a**, Total number of new lesions by CT during period 2 relative to period 1 (mITT analysis set). **b**, Total number of new lesions by ^18^F-NaF PET during period 2 relative to period 1 (mITT analysis set). **c**, Mean total volume of new lesions as assessed by CT in period 2 relative to period 1 (mITT analysis set). **d**, TLA of new lesions by 18F-NaF PET in period 2 relative to period 1 (mITT analysis set). **e**, Percent of patients with new lesions by CT during period 2 relative to period 1 (mITT analysis set). **f**, Percent of patients with new lesions by ^18^F-NaF PET during period 2 relative to period 1 (mITT analysis set). *P* values were generated from a Wilcoxon signed-rank test (**a**–**d**) and a McNemar’s test (**e**,**f**). *P* values were not adjusted for multiple testing; however, a hierarchical testing strategy was prespecified before analysis of the data.

Of the 43 patients in the AHO population who completed period 1, all entered and 42 completed period 2. The main statistical comparisons were based on within-group comparisons for patients randomized in period 1 to placebo and crossed over to receive garetosmab in the COVID-19 mITT population (*n* = 22). For period 2, all primary and key secondary end points achieved statistical significance (two-sided *P* < 0.1) in the predefined hierarchy for period 2 (Table 3b). The total number of new lesions measured by CT for patients who crossed over from placebo to garetosmab was reduced by 100% relative to period 1 (0 versus 22, respectively; *P* = 0.0039) and by 95% as measured by PET (1 versus 23, respectively; *P* = 0.0039; Fig. 3a,b and Table 3b). Mean new lesion volume and activity (secondary end points) were significantly lower for period 2 than period 1 (volume, 0.05 cm^3^ versus 9.29 cm^3^, respectively, *P* = 0.0039; activity, 13.20 g versus 204.45 g, respectively; *P* = 0.0273; Fig. 3c,d). This reduction was also reflected in the percentage of patients who developed new lesions, which was significantly lower during period 2 compared to period 1 by CT (0% versus 40.9%, respectively; *P* = 0.0027) and PET (4.5% versus 40.9%, respectively; *P* = 0.0047; Fig. 3e,f). The additional protocol-defined secondary end points that were not in the hierarchical testing sequence are reported in Supplementary Table 6b.

**Extended Data Fig. 8:**
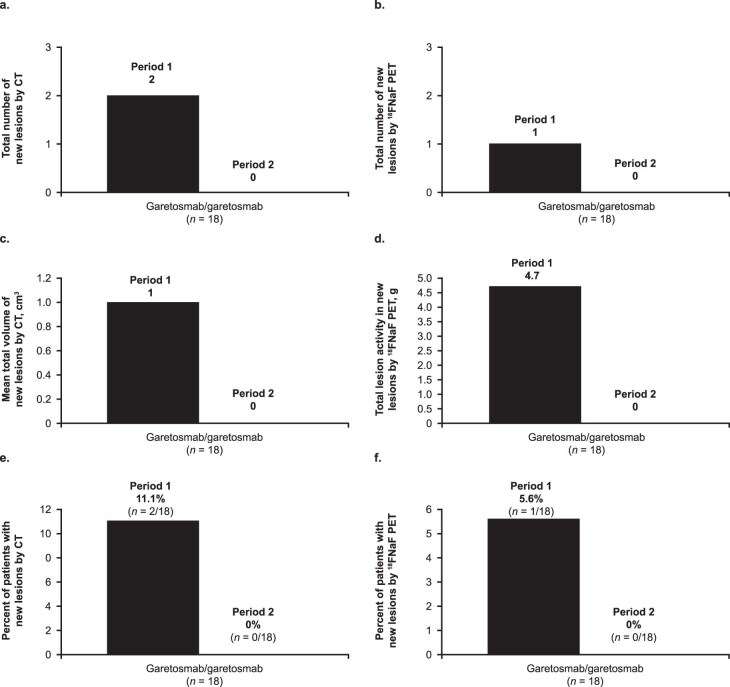
Durability of garetosmab effect (period 1 through period 2): New HO lesions and volume by CT and PET. Effect of garetosmab in period 2 relative to period 1 on the **a**, **b**, total number of new HO lesions, **c**, mean total volume of new HO lesions **d**, TLA of new HO lesions and **e**, **f**, percent of patients with new lesions, as assessed by quantitative imaging in patients originally randomized to garetosmab in period 1. CT, computed tomography; HO, heterotopic ossification; PET, positron emission tomography; TLA, total lesion area.

For patients originally randomized to garetosmab and who remained on garetosmab in period 2, garetosmab’s efficacy in preventing new HO lesions was maintained (0 new lesions by CT and PET in period 2 versus two by CT and one by PET in period 1; Extended Data Fig. 8). Although the two lesions identified by CT in period 1 were still detectable in period 2, the volume remained stable (5.19 cm^3^ to 5.00 cm^3^) or substantially decreased (13.69 cm^3^ to 2.37 cm^3^). Additionally, the percentage of patients with new lesions was 0% for period 2 relative to period 1 by PET and CT versus 5.6% by PET and 11.1% by CT for period 1 relative to baseline (Extended Data Fig. 8). The new lesion volume was 0 cm^3^ for period 2 versus 1 cm^3^ for period 1 and the TLA was 0 g versus 4.7 g, respectively (Extended Data Fig. 8). The proportion of patients with new flare-ups by patient diary was 22.2% (4 of 18) in period 2 and 33.3% (6 of 18) in period 1 and by investigator AE report was 5.6% (1 of 18) and 11.1% (2 of 18), respectively (Supplementary Table 6).

Regarding flare-ups, for patients crossing over from placebo to garetosmab after week 28, the proportion with new flare-ups was significantly lower in period 2 compared to period 1 as reported by both patients (13.6% versus 68.2%, respectively) and investigators (13.6% versus 45.5%, respectively; Supplementary Table 9 and Extended Data Fig. 7c,d). The total number of new flare-ups by patient diary was 11 in period 2 versus 31 in period 1 and by investigator was 4 and 22, respectively (Extended Data Fig. 7e,f).

### Open-label extension results

During the subsequent open-label extension period, no new HO lesions at week 76 were observed in patients crossing to garetosmab after week 28 (*n* = 17; COVID-19 modified ITT; prespecified), whereas one new lesion was observed by both PET and CT at 76 weeks among patients continuing garetosmab since baseline (*n* = 15). Overall, treatment with garetosmab resulted in a sustained and pronounced effect in preventing new HO lesions from forming up to 76 weeks (the time of the last imaging scan assessment in the study). Thirty-four (77%) patients continued in the study after the open-label extension.

## Discussion

LUMINA-1 assessed the safety and efficacy of garetosmab, an activin A-blocking monoclonal antibody, in patients with FOP. Based on data from a genetic model of FOP in rodents^14,15^, it was initially hypothesized that blockade of activin A would lead to a reduction in the activity (as assessed by ^18^NaF PET) and growth (as assessed by volumetric CT) of pre-existing HO lesions as well as to prevent the formation of new HO. In the initial randomized, placebo-controlled portion of the trial (period 1), it was learned that garetosmab treatment did not result in the robust regression or decreased activity of pre-existing HO lesions over 28 weeks. It was noted, however, that pre-existing HO lesions did not appreciably grow over this period (even on placebo). Moreover, consistent with the preclinical evidence, garetosmab markedly suppressed the occurrence and growth of new HO lesions. As a result of these observations, the hypothesis and testing for the open-label portion of the trial (period 2) was revised to primarily assess the impact of garetosmab on the formation of new lesions as patients crossed over from placebo to active treatment. Subsequent formal statistical testing confirmed near-complete blockade of new HO lesions.

Flare-ups, while the pathogenesis is not well understood, create a substantial burden for patients with FOP; they are often painful and debilitating and may require the use of corticosteroids in high doses or over prolonged periods of time. Flare-ups were also noted to be reduced by garetosmab, additionally suggesting an activin A mechanism for this phenotype.

AEs associated with garetosmab included epistaxis, madarosis and a composite of skin and soft-tissue infections. Investigation of coagulation function and BMP9 levels did not provide definitive insights into the potential mechanisms of epistaxis. These AEs had not been observed in two previous early-phase studies of garetosmab conducted in healthy volunteers (Clinicaltrials.gov identifiers NCT02870400 and NCT02943239), nor were they noted in a phase 1 study combining garetosmab with an anti-myostatin antibody in post-menopausal females^25^. They also were not observed in clinical trials in healthy volunteers or non-FOP patients with a monoclonal antibody targeting the activin receptor type-2B, downstream of activin A^26,27^. Signals suggestive of an underlying bleeding or infection issue also did not manifest in preclinical studies^14,15^. It may be possible that these garetosmab-related AEs represent a unique drug–disease interaction and occur specifically in the context of altered activin A signaling in FOP.

Five fatal events occurred after 8–16 (median, 15) doses during the open-label periods of the study. This number is high for a small study. Each of the deaths was assessed by investigators as being unrelated to treatment and there were no clear patterns to link them with garetosmab nor the mechanism of activin A inhibition. Four out of the five patients who died had particularly advanced disease with high CAJIS scores, while the fifth patient fell down a flight of stairs suffering grievous injuries. While no common pathogenetic mechanism of these deaths could be determined, they occurred during the open-label periods. There are published reports of mortality in FOP; however, there are no published reports of annualized rates of death in this community^15^ contributing to challenges in interpretation. With this in mind, post-hoc assessment of safety data by all the authors concluded that (1) epistaxis events were related to garetosmab; (2) there was no apparent association between epistaxis and patient deaths; and (3) causes of death appeared consistent with known causes of death and the life expectancy for patients with FOP who were of similar age and disease^10^.

Limitations of this phase 2 study in ultra-rare patients with FOP include the relatively small sample size and duration of treatment with a placebo control (28 weeks), before giving all patients garetosmab for the following 28 weeks or longer. The fatalities described above all occurred during the open-label, non-placebo-controlled portion of the study, making definitive assessment of safety and the causality of the deaths highly challenging. With respect to the assessment of the efficacy of garetosmab, the primary placebo-controlled end point, TLA, a reflection of the size and activity of existing HO lesions, was not met. As this was the first prospective, placebo-controlled and systematic application of ^18^F-NaF PET/CT in FOP, unexpected but important insights regarding the lack of growth of pre-existing HO lesions were gained in period 1. This required an unusual but necessary protocol amendment so that a more appropriate hypothesis could be prospectively tested in period 2 and beyond. The primary end point utilized in period 2 was a within-group comparison for patients crossing over to garetosmab treatment (comparing the number of HO lesions formed in period 2 to period 1). This approach is not considered as robust as one utilizing a fully independent, placebo-controlled cohort. Nevertheless, despite these limitations, the results were compelling.

Together with previously published preclinical data in murine models of FOP, the data generated during LUMINA-1 demonstrate that activin A is a required ligand for HO in FOP and provides strong evidence that inhibition of activin A using garetosmab is a promising disease-modifying therapy with the ability to not only block HO but also reduce the number and severity of painful, soft-tissue inflammatory flare-ups, further alleviating the burden of disease. As the most profound effects were on prevention of new HO lesions, the greatest utility of garetosmab treatment may be early in the course of disease, before substantial disability has accrued. Recently, palovarotene was approved by the US Food and Drug Administration (FDA) for the treatment of FOP.

Garetosmab treatment has demonstrated substantial and durable reduction of new heterotopic bone lesion formation and soft-tissue inflammatory flare-ups in adults with FOP. Although a definitive link between garetosmab and the five deaths was not established in this population with advanced disease, the number of deaths was relatively high for this small study; therefore, the benefit–risk profile of garetosmab is currently being further evaluated in the phase 3 OPTIMA trial (NCT05394116) and pediatric studies are anticipated. Garetosmab may provide a therapeutic option in this ultra-rare, severely debilitating, life-threatening disease.

## Methods

### Compliance and trial oversight

Patient safety and welfare were monitored by an Independent Data Monitoring Committee. This study was conducted in accordance with the 2013 Declaration of Helsinki, the International Council for Harmonization guidelines for Good Clinical Practice and SAGER guidelines. All patients provided written, informed consent.

LUMINA-1 (NCT03188666) was conducted at 11 sites in eight countries. The full protocol is available online. The trial was approved by the following institutional review boards: University Health Network, Toronto, Ontario, Canada; Comité de Protection des Personnes, Paris, France; Comitato Etico Regione Liguria, IRCCS Ospedale Policlinico, San Martino, Genoa, Italy; Science Committee AMS, VUmc, Amsterdam, The Netherlands; METC VUmc BS7, Amsterdam, The Netherlands; Komisja Bioetyczna Uniwersytetu Rzeszowskiego, Rzeszów, Poland; Comité de Ética de la Investigación con Medicamentos del Hospital Universitario Ramón y Cajal, Madrid, Spain; London Central Research Ethics Committee, Manchester, UK; University of Pennsylvania, Office of Regulatory Services, Philadelphia, Pennsylvania, USA; Mayo Clinic Institutional Review Board, Rochester, Minnesota, USA; Vanderbilt University, Nashville, Tennessee, USA.

### Study design

LUMINA-1 (NCT03188666) was a phase 2, randomized, double-blind, placebo-controlled study evaluating the safety, tolerability and effects on HO of i.v. garetosmab 10 mg kg^−1^ Q4W. The study was conducted at 11 sites in eight countries across North America and Europe; the first patient was enrolled on 26 February 2018, the last patient was enrolled on 25 February 2019 and the last patient completed the study on 16 September 2021. ^18^F-NaF PET and whole-body, low-dose X-ray CT^28^ were used to measure and track HO. The study consisted of a 28-d screening/baseline period followed by a 28-week randomized, double-blind, placebo-controlled period (period 1), a 28-week open-label treatment period (period 2) and a subsequent open-label extension from week 56 to the end of the study (Supplementary Fig. 2). The primary analysis was conducted when all patients completed period 1. Further predefined analyses were conducted at the end of period 2.

Six protocol amendments were implemented during the conduct of the study: amendment no. 1, Addition of open-label extension period; amendment no. 2, Addition of *ACVR1* gene sequencing and inclusion of patients with non-classical mutations; amendment no. 3, Modifications to selection criteria, testing and analyses due to AEs of epistaxis; amendment no. 4, Modification of the statistical testing hierarchy based on mutation type; amendment no. 5, Adjustment of study medication formulation details; and amendment no. 6, Adaptation of study procedures due to the COVID-19 pandemic and a prospective restatement of the hypothesis and end points for period 2. Amendments are detailed in Supplementary Table 10.

### Patient inclusion and exclusion criteria

The study population included male and female patients aged 18–60 years at screening with a clinical diagnosis of FOP (based on findings of congenital malformation of the great toes, episodic soft-tissue swelling and/or progressive HO) and a history of FOP disease activity (defined as pain, swelling, stiffness and other signs/symptoms associated with FOP flare-ups; or worsening of joint function, or radiographic progression of HO (increase in site or number of HO lesions) with/without being associated with flare-up episodes) within 1 year of screening, plus documentation of an *ACVR1* mutation. In addition, patients had to be willing and able to attend and comply with study visits and to undergo PET and CT imaging procedures.

Patients were excluded if they met the following exclusion criteria:

- Relevant concomitant illness or history of relevant illness such as but not limited to cardiac, renal, rheumatologic, neurologic, psychiatric, endocrine, metabolic or lymphatic disease.
- Previous history or diagnosis of cancer.
- Used bisphosphonate therapies within 1 year of screening, as these medications alter bone metabolism and would confound the primary efficacy analysis.
- Concurrent participation in another interventional clinical study, or a non-interventional study with radiographic measures or invasive procedures (for example, collection of blood or tissue samples). Participation in the FOP Connection Registry or other studies in which participants complete study questionnaires was allowed.
- Treatment with another investigational drug, denosumab, imatinib or isotretinoin in the last 30 d or within five half-lives of the investigational drug, whichever was longer.
  - To address a potential risk of embryotoxicity or male reproductive organ toxicity, the protocol also excluded pregnant or breastfeeding females, as well as males and females of child-bearing potential who were unwilling to practice highly effective contraception.
  - To address a potential risk of epistaxis identified during the study in period 1, the following exclusion criteria were added (protocol amendment no. 3):
- Patients on concomitant antiplatelet therapy (for example, clopidogrel), anti-coagulants (for example, warfarin, heparin, factor Xa inhibitor or thrombin inhibitors) in the last 30 d or within five half-lives of the therapy, whichever was longer. Low-dose acetylsalicylic acid (aspirin) was acceptable.
- Patients with a history of severe, non-traumatic bleeding requiring transfusion or hospitalization for hemodynamic compromise.
- Patients with a known pre-existing medical history of a bleeding diathesis (for example, hemophilia A, von Willebrand’s factor deficiency, platelet count ≤20 × 109 l^−1^).
- Detailed inclusion and exclusion criteria are provided in the full protocol available online.
- Patients on concomitant antiplatelet therapy (for example, clopidogrel), anti-coagulants (for example, warfarin, heparin, factor Xa inhibitor or thrombin inhibitors) in the last 30 d or within five half-lives of the therapy, whichever was longer. Low-dose acetylsalicylic acid (aspirin) was acceptable.
- Patients with a history of severe, non-traumatic bleeding requiring transfusion or hospitalization for hemodynamic compromise.
- Patients with a known pre-existing medical history of a bleeding diathesis (for example, hemophilia A, von Willebrand’s factor deficiency, platelet count ≤20 × 109 l^−1^).
- Detailed inclusion and exclusion criteria are provided in the full protocol available online.

### Randomization and masking

Enrolled patients were randomized (1:1) to receive garetosmab 10 mg kg^−1^ Q4W, as previously assessed^25^ or placebo in period 1, according to a central randomization scheme. Block randomization was conducted using an interactive response technology provided to the designated study pharmacist or qualified designee. Randomization was stratified by presence/absence of baseline active HO lesions, sex and mutation type. All PET–CT scans were reviewed by two independent readers and an adjudicator; all three were blinded to treatment assignment.

### Procedures

Patients were assigned to receive garetosmab 10 mg kg^−1^ or placebo Q4W for 28 weeks (period 1). After week 28, all patients continued or were crossed over to garetosmab 10 mg kg^−1^ Q4W (period 2). At the conclusion of period 2, patients were given the option to continue on garetosmab in an open-label extension. Whole-body PET–CT scans were acquired to identify pre-existing lesions at baseline, identify new HO lesions and measure volume changes in pre-existing and new HO lesions. Baseline imaging with PET–CT was performed within 7 d before initial study drug administration and at weeks 8, 28, 56 and 76 (Supplementary Fig. 2).

### Outcomes

#### Safety

The primary end point for period 1 was the incidence and severity of AEs, which included both those not present at baseline and those that were an exacerbation of a pre-existing condition. A full safety profile to the end of study was descriptively reported.

#### Period 1 efficacy

The prespecified primary end point for efficacy was the effect of garetosmab versus placebo on the TWA of the percentage change from baseline in TLA by PET; TLA is considered proportional to the deposition rate of bone mineral into actively forming HO lesions. The next end point in the hierarchy was to assess the percentage change in the total volume of HO lesions by CT in period 1 relative to baseline. The last end point in the hierarchy was the TWA change from baseline in daily pain due to FOP as measured using the daily NRS over 28 weeks in AHO and AHOC. A full list of secondary and exploratory end points is provided in Supplementary Table 11. Exploratory end points in period 1 included the percentage of patients with flare-ups as assessed by patient diary and post-hoc analyses included investigator reported flare-ups.

#### Period 2 efficacy

Based on the outcomes of period 1, the prespecified primary end point for efficacy for period 2 was prospectively changed to the number of new lesions in patients crossing over from placebo to garetosmab as assessed by CT. Additionally, we assessed efficacy at week 56 relative to week 28 in total volume of new HO lesions by CT, the number of new lesions by PET, TLA by PET in new HO lesions and percent of patients with new lesions by CT and PET. A full list of secondary and exploratory end points is provided in Supplementary Table 11.

### Imaging rationale

Imaging by ^18^F-NaF PET identifies new bone formation and mineralization through the accumulation of ^18^F as it substitutes hydroxyl groups in newly formed hydroxyapatite^29^. The US FDA-approved imaging by ^18^F-NaF PET has been widely used to detect and quantify changes in abnormal osteogenic activity in several bone pathologies such as Paget’s disease, ankylosing spondylitis and osteoblastic bone metastases^30^. In patients with FOP, ^18^F-NaF PET has been used to identify HO lesions with a high PET signal, which also showed growth by CT over a period ranging from 5 to 20 months; lesions with a low PET signal, consistent with bone remodeling in the normotopic skeleton, showed no growth by CT over the same period^22^. CT allows identification and differentiation of HO lesions from normal skeletal bone and quantification of the volume of heterotopic bone. Whole-body volumetric measurement of HO by CT is recommended as a clinical end point in FOP studies by the International Clinical Council on FOP^31^.

In LUMINA-1, ^18^F-NaF PET/CT was used to identify pre-existing lesions at baseline, identify new HO lesions, measure osteogenic activity of bone lesions, differentiate mineralizing lesions from mature HO and measure volume changes in pre-existing and new HO lesions. Baseline imaging with PET–CT was performed at most 7 d before study drug administration and at subsequent time points (Supplementary Fig. 2).

A previous retrospective study demonstrated the utility of PET in FOP patients to identify active HO lesions by demonstrating that, over a period of 5–20 months, only those lesions with high PET signal showed growth by CT, whereas lesions with low PET signal (equivalent to that of the normotopic skeleton) showed no growth by CT^22^. Furthermore, at the time of LUMINA-1’s design, it was unknown whether a sufficient number of new HO lesions would arise over the 28-week interval (period 1) to rely on them alone for assessing the efficacy of garetosmab. Therefore, it was hypothesized that PET presented the most sensitive imaging modality to quantify total change in HO activity, whereas CT would enable detection of changes in volume of any HO lesion during period 1.

### Imaging acquisition and read procedures

Whole-body PET–CT scans were acquired. PET–CT acquisition and reconstruction parameters within prespecified ranges were defined for each patient at the baseline scan and kept constant throughout the study. All PET–CT scans were transferred to a contract research organization for centralized quality control and review by two independent readers and an adjudicator; all three were blinded to treatment assignment. Based on preclinical data, we hypothesized that HO lesions which showed the highest uptake of ^18^F-NaF on a baseline PET image would be most likely to show rapid growth when untreated and would be inhibited from growing by garetosmab treatment^15^. HO lesions showing high ^18^F-NaF uptake were defined as being active, with a maximal standardized uptake value (SUV_max_) ≥ 3 times that of the mean standardized uptake value (SUV_mean_) of a normotopic reference region in the supra-acetabular area of the pelvis. The SUV_mean_ is defined as the mean decay-corrected activity concentration (r) within a given region of interest of a PET image divided by the total injected radioactivity dose (A_0_) normalized by body mass (M_0_):

$$\left({{SUV}}_{{mean}}=\frac{r}{{A}_{0}/{M}_{0}}\right).$$

SUV_max_ is the SUV of the most intense voxel within a region of interest (for example, an HO lesion) in a PET image. Metabolic volume of an HO lesion is the sum volumes of voxels with an SUV above a threshold defined as 40% of the SUV_max_ of that lesion. This thresholding approach was chosen for its simplicity and was based on previous published experience in tumor delineation using ^18^F-FDG PET, which has similarly high uptake in tumors as in active HO lesions in FOP patients^32^. Readers were instructed to manually exclude normotopic bone or other regions they deemed not appropriate to include within the metabolic volume of an HO lesion.

From each patient’s baseline PET–CT images, readers selected up to seven candidate active HO pre-existing lesions. Subsequently, an adjudicator chose up to seven lesions from this initial pool as the set of pre-existing lesions to be followed and quantified by both readers, beginning with the lesion with the greatest SUV_max_ and continuing in a descending order of signal magnitude. The total number of pre-existing lesions was limited as a compromise between the need to select a large enough pool of representative active lesions, and operational limitations around the delineation and quantification of these lesions in PET and CT images. Pre-existing lesions thus identified were followed throughout the study and included in the analysis of inhibitory effect on HO formation by garetosmab versus placebo. A decrease in volumetric growth was measured by CT in active pre-existing lesions.

Appearance of new lesions was assessed by readers post-baseline using PET–CT scans. A third independent blinded reader (the adjudicator) performed forced adjudication when there were discrepancies between the two independent readers in the assessment of new lesions. Readers were blinded to the adjudication. New lesions developing post-baseline as identified by PET were required to be active. New lesions identified by CT alone, consistent with HO location and morphology, were required to have a density >200 HU (well above the value of ~50 HU of soft tissue to increase the certainty of bone identification, but unlikely to exclude cortical HO bone with ~800 HU or greater) with a volume ≥1 cm^3^ (close to the minimum HO lesion volume that could be reliably measured in a low-dose CT scan).

Changes in volumetric growth were assessed by CT in active, pre-existing lesions. Readers independently assessed PET SUV_max_, SUV_mean_, peak SUV and total metabolic volume of each HO lesion. TLA, a measure of patient-level overall burden of growing and actively mineralizing HO lesions, was calculated as the sum of the product of pre-existing and new HO lesion’s SUV_mean_ and metabolic volume at each time point.

### BMP9 methods

Total soluble BMP9 concentrations in human serum were measured using an enzyme-linked immunosorbent assay, which uses a mouse anti-human BMP9 monoclonal antibody as the capture reagent and recombinant BMP9 as the standard. Captured soluble BMP9 is detected using a biotinylated goat anti-human BMP9 polyclonal antibody followed by streptavidin conjugated with horseradish peroxidase. The lower limit of quantitation of the assay is 31.3 pg ml^−1^ in neat human serum.

### Statistics and reproducibility

#### Sample size and multiplicity strategy

The sample size estimation for at least 24 patients (12 patients per treatment group) with active HO at baseline and classic ACVR1(R206H) mutation was based on statistical considerations for the following efficacy end points: percent change from baseline in (1) TLA by ^18^F-NaF PET over 28 weeks; (2) total volume of HO lesion by CT at week 28; and (3) ^18^F-NaF SUV_max_ at week 8. Accounting for a 20% dropout rate at week 28, the sample size would yield approximately ten patients per treatment group for week-28 analyses. This sample size was estimated to provide 80% power at a two-sided 0.05 significance level for allowing the detection of an observed treatment difference in the order of 57%, 65% and 40% reduction in the TLA by ^18^F-NaF PET, the total volume of HO lesion by CT and the ^18^F-NaF SUV_max_, respectively, based on other bone diseases, including FOP^22^ and modeling in FOP mice^14,15,33^.

In period 1, testing of the primary and key secondary efficacy end points followed a hierarchical testing procedure to address multiplicity at an overall two-sided α = 0.05 significance level. Testing of the key secondary efficacy end points would follow a hierarchical testing sequence only if statistical significance was established for all primary end points. No further adjustments were made for other secondary and exploratory end points, for which nominal *P* values were to be provided for descriptive purposes only. Safety outcomes were analyzed using descriptive statistics.

In period 2, the evaluation of these prospectively specified redefined primary and key secondary end points relating to new heterotopic bone formation warranted new analyses, which were independent from that of period 1 and required their own overall type I error rate of 10% (α of 0.1). To control the type I error rate at 0.10 for the primary and key secondary null hypotheses in period 2, a hierarchical testing procedure was applied at a two-sided 10% significant level as detailed. No further adjustments were made for other secondary and exploratory end points in period 2, for which estimates, 95% CI and/or nominal *P* values were to be provided for descriptive purposes. Safety outcomes were analyzed using descriptive statistics.

#### Missing data strategy

For the primary end point, the area under the curve (AUC) of percent change from baseline in TLA by ^18^F-NaF PET over 28 weeks was calculated for each patient. If both imaging scans at weeks 8 and 28 were missing, then mean percent changes of the placebo group at week 8 and 28 were used for imputation. If the imaging scan for only week 8 was missing, linear interpolation of percent change between the baseline and week 28 was used to calculate the AUC. If the imaging scan for week 28 was missing, percent change at week 8 was carried forward to week 28 for the calculation AUC.

Briefly, for patients whose week 56 scans were delayed or missed due to the pandemic, the first available scan after the week 28 scan was used to impute the week 56 data in the primary analyses. In the absence of a PET–CT scan, data from the first available CT-only scans after the week 28 scan were used to analyze the primary end point and other secondary imaging end points as applicable. For missing week 56 scans solely due to the COVID-19 pandemic in patients who crossed over to garetosmab from placebo, the assumption of ‘missing completely at random mechanism’ was reasonable.

#### Analysis populations

In period 1, the study populations included a baseline AHO of all randomized patients with at least one active HO lesion at baseline. Patients with active HO lesions (lesions with active mineralization) were defined as those patients at baseline who had at least one HO lesion demonstrating uptake of ^18^F-NaF PET of at least three times that of normal reference bone (supracetabular bone) as assessed by central review. All randomized patients had active disease at baseline, so the AHO and full analysis sets are identical. The statistical analysis plan (Supplementary information) also specified a baseline AHOC *ACVR1*^R206H^ mutation analysis set; only two randomized patients had atypical *ACVR1* mutations. The safety analysis set included all randomized patients who received any study drug.

In period 2, the COVID-19 pandemic caused delays in dose administration, PET–CT scan collection and PET tracer availability for some patients (*n* = 4). To mitigate confounding effects on study outcomes, period 2 analyses were based on a COVID-19 mITT analysis, defined as all patients with active HO at baseline who received treatment in period 2 and for whom at least one post-week-28 scan was collected, with the period between consecutive garetosmab doses being <9 weeks before the first post-week-28 scan. For the analysis of week 56 and week 76, these criteria excluded a couple of patients. Statistical methodology amendments are detailed in Supplementary Table 10.

### Statistical analyses

For the period 1 primary analysis, the TWA percent change from baseline in TLA ^18^F-NaF PET over 28 weeks was analyzed in the baseline AHO analysis set and the baseline active HO classic ACVR1(R206H) mutation (AHOC) analysis set populations using an analysis of covariance model. The difference in LS mean change from baseline, 95% CI and *P* value were provided from the model to compare garetosmab with placebo. Percent change from baseline in the total volume of HO lesions as assessed by CT at week 8 and 28 weeks was analyzed in AHO analysis set and AHO analysis set using an MMRM model. The model contains treatment, sex, *ACVR1* mutation type (classic and non-classic), visit (weeks 8 and 28), baseline total volume and treatment-by-visit interaction. An unstructured covariance was used to account for within-patient correlation between time. Difference in LS mean change from baseline, the corresponding 95% CI and the *P* value were provided from the MMRM model for comparison of the garetosmab group against the placebo group. TWA (standardized AUC) change from baseline in daily pain due to FOP, as measured using the daily NRS over 28 weeks in AHO and AHOC, was analyzed using the ANCOVA model.

For the period 2 primary analysis, the Wilcoxon signed-rank test was used to compare the number of new HO lesions as assessed by CT at week 56 (relative to the week 28 scan) with the number of new HO lesions at week 28 (relative to the baseline scan) in patients who crossed over from placebo to garetosmab. The estimate and 95% CI of the rate of new HO lesions at week 56 and that of the rate ratio (comparing period 2 to period 1) were based on a negative binomial model with repeated measures and using a generalized estimating equation. Safety outcomes were analyzed using descriptive statistics. To estimate the difference between period 2 and period 1 in total volume associated with new HO lesions, a MMRM model was implemented. The response variable was the total volume of new lesions at week 56 (relative to the week 28 scan) and at week 28 (relative to the baseline scan). The model included visit (week 28 and week 56) and the total volume by CT at baseline as a covariate. An unstructured covariance was used to account for within-patient correlation between visits. LS mean of differences in total volume of new lesions and the corresponding 95% CI are provided for comparison for week 56 against week 28. Similar methods were implemented for the corresponding PET-related end points. For the proportion of patient-related end points, the number and percent of patients who responded at week 56 relative to week 28 are provided for the placebo/garetosmab group (COVID-19 mITT set). In general, the within-group comparison used a McNemar’s test in the placebo/garetosmab group. A logistic regression model with repeated measures was used to estimate the odds ratio and 95% CI to compare period 2 and period 1 using the GEE. The model included visit (week 28 and week 56) and the baseline total number of lesions by CT (or PET) as a covariate. An unstructured covariance was used to account for within-patient correlation between time points.

### Reporting summary

Further information on research design is available in the Nature Portfolio Reporting Summary linked to this article.

## Online content

Any methods, additional references, Nature Portfolio reporting summaries, source data, extended data, supplementary information, acknowledgements, peer review information; details of author contributions and competing interests; and statements of data and code availability are available at 10.1038/s41591-023-02561-8.

### Supplementary information

Supplementary Information

Reporting Summary

## Figures and Tables

**Extended Data Fig. 6:**
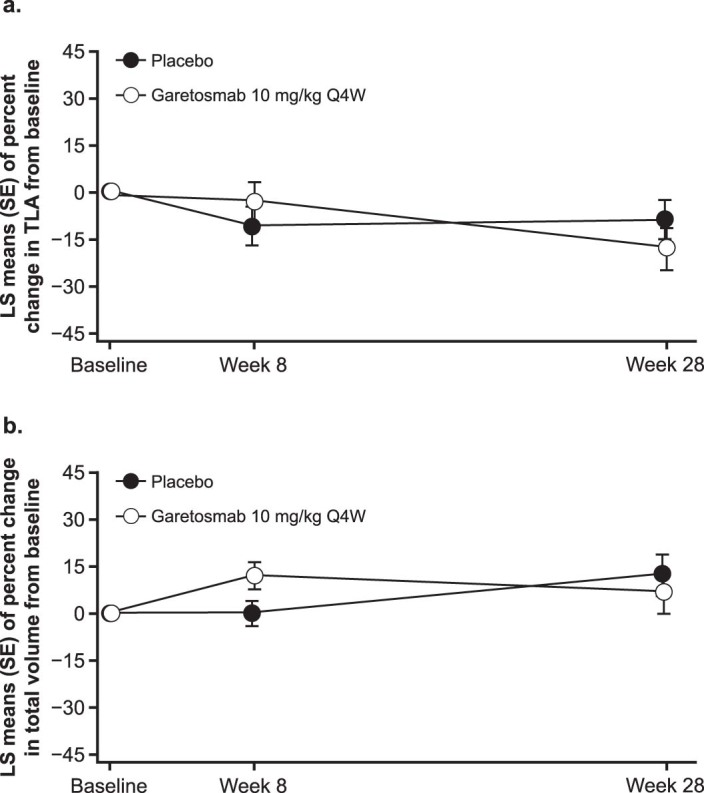
Percent change from baseline in total lesion activity by ^18^F-NaF PET and total volume of HO lesions by CT over time for pre-existing lesions (AHO). **a**, LS mean percent change from baseline in TLA by ^18^F-NaF PET over 28 weeks. **b**, LS mean percent change from baseline of total HO lesion volume assessed by CT. LS means and SEs were estimated using a MMRM. This model included fixed categorical effects of treatment group, gender, time point, treatment-by-time point interaction, as well as the continuous fixed covariates of baseline value. ^18^F-NaF PET, fluorine-18-labeled sodium fluoride positron emission tomography; AHO, active heterotopic ossification analysis set; BL, baseline; CT, computed tomography; HO, heterotopic ossification; LS, least squares; MMRM, mixed-effect model for repeated measures; Q4W, every 4 weeks; SE, standard error.

## Data Availability

Qualified researchers may request access to study documents that support the methods and findings reported in this manuscript. Individual anonymized patient data will be considered for sharing once the product and indication has been approved by major health authorities (for example, US FDA, European Medicines Agency and the Pharmaceuticals and Medical Devices Agency), if there is legal authority to share the data and there is not a reasonable likelihood of patient re-identification. Requests should be submitted to https://vivli.org/ (the typical response time is 6–12 months).

## Extended data

**Extended Data Table 1:** Case summaries of patients who died

Case 1	A 26 to 30-year-old^a^ with a CAJIS score of 16/30 with ankylosis of hips, knee and shoulders had an SAE of severe head injury (resulting from a fall) in the open-label extension, which was immediately followed by death. The patient had been randomized to garetosmab in period 1 and received 16 doses of garetosmab by the time of the event. Prior to this event, the patient had severe gait impairment and required aid to perform daily activities. An autopsy showed major head trauma, with a laceration of the scalp in the vertex region and periorbital hematoma on an external examination. Internal examination revealed a diastatic skull fracture together with diffuse subdural and subarachnoid hemorrhages, as well as multiple fractured ribs^1^. The cause of the death was reported as being due to severe head and brain trauma due to a fall, in the setting of a patient with diffuse rigidity and walking disability.
Case 2	A 41 to 45-year-old with a CAJIS score of 28/30 commensurate with complete ankylosis in the spine, shoulders, hips, knee joints and locked jaw experienced a hemorrhagic stroke in the open-label extension. The patient had been randomized to garetosmab in period 1 and received 16 doses of garetosmab by the time of the event. During hospitalization, the patient’s condition deteriorated and the patient died. The cause of death was reported as a massive hemorrhagic stroke to the deep structures of the brain leading to acute respiratory failure. No autopsy was performed at the request of the patient’s family. This patient had poorly controlled arterial hypertension and had experienced two AEs of worsening hypertension during the study. Review of the head CT images (captured on the day of the hospitalization and a couple of days after the event), later performed by two independent radiologists, reported findings suggestive of chronic hypertensive effects and agreed that the hemorrhage was typical for a hypertensive bleed and not due to a ruptured cerebral vascular malformation.
Case 3	A 36 to 40-year-old patient with a CAJIS score of 30/30 with complete ankylosis of the spine, shoulder, hip and knee joints bilaterally as well as major jaw stiffness experienced a fatal SAE of intestinal obstruction during period 2. The patient had been randomized to garetosmab in period 1 and received 14 doses of garetosmab by the time of the event. The patient was hospitalized for constipation, non-specific abdominal pain, vomiting and fever. Imaging studies confirmed a mechanical obstruction and laboratory tests showed elevated inflammatory markers. They were treated with enemas, nasogastric tube and broad-spectrum antibiotics. The patient’s condition initially improved, but rapidly deteriorated following discharge to a nursing home facility resulting in death. An autopsy was not performed. The patient had experienced an episode of intestinal obstruction during study period 1 which also had led to an SAE (hospitalization) and resolved with clinical procedures, without the need of surgical intervention.
Case 4	A 31 to 35-year-old patient with a CAJIS score of 26/30 with almost complete ankylosis of the neck, thoracic and lumbar spine, shoulders, elbows, hips and knees, and with movement only in wrists and ankles, experienced a fatal SAE of traumatic splenic rupture following a fall in the open-label extension. The patient had been randomized to placebo (seven doses) in period 1 and received eight doses of garetosmab in the open-label period by the time of the event. The patient was ‘frozen’ in a flat horizontal position, though with assistance they could switch to a vertical position, with limited balance. A surgical procedure was started, but after a difficult intubation the patient experienced cardiac arrest and died. An autopsy was not performed, and the investigator assessed this SAE as more likely related to the severity of the underlying disease and the trauma.
Case 5	A 36 to 40-year-old patient with a CAJIS score of 19/30 experienced sudden death in the open-label extension. The patient had been randomized to placebo (seven doses) in period 1 and received 15 doses of garetosmab in the open-label period by the time of the event. The patient’s past medical history was indicative for hypothyroidism, brain cavernoma hemorrhage (around the age of 20 years), mild renal insufficiency and an uncommon skin condition known as pigmented purpuric dermatosis. A preliminary macroscopic post-mortem exam noted gross right lung hemorrhage, but this was later determined to be incorrect. A full autopsy report ruled out any lung hemorrhage or acute bleeding event and determined that the most likely cause of death was aspiration pneumonia. After consultation with a second pathologist, the final cause of death was considered sudden cardiac death, with additional findings of extensive granulomatous inflammation most likely attributable to chronic ongoing aspiration of foreign material.

^a^To protect patient identity, age ranges for individuals are given rather than specific ages.

^1^Bolcato, V. et al. New insights on fibrodysplasia ossificans progressiva: discussion of an autoptic case report and brief literature review. *Intractable* *Rare* *Dis*. *Res*. **10**, 136–141 (2021).

AE, adverse event; CAJIS, Cumulative Analog Joint Involvement Scale; CT, computed tomography; SAE, serious adverse event.
