# Supplementary material for: Garetosmab in fibrodysplasia ossificans progressiva: a randomized, double-blind, placebo-controlled phase 2 trial
Source: Nat Med. 2023 Sep 28;29(10):2615–24. doi: 10.1038/s41591-023-02561-8 (PMC10579054; doi:10.1038/s41591-023-02561-8)
Supplement: Supplementary file 1 — Supplementary Information [file 41591_2023_2561_MOESM1_ESM.pdf]

nature medicine

Article

https://doi.org/10.1038/s41591-023-02561-8

Garetosmab in fibrodysplasia ossificans progressiva: a randomized, double-blind, placebo-controlled phase 2 trial

In the format provided by the authors and unedited

Table of contents

Study sites and investigators 3

Table S1. TEAEs that occurred in ≥10% of the garetosmab 10 mg/kg Q4W group in the double-blind period of the study (Period 1)[a,b] 5

Table S2. Skin and soft tissue infection TEAEs[a,b] 8

Table S3. SAEs that occurred in the double-blind period of the study (Period 1)[a,b] 10

Table S4. Bleeding TEAEs 11

Table S5. SAEs that occurred in the open-label period[a,b] 12

Table S6. Summary of all protocol pre-specified secondary and exploratory endpoints for Period 1 and Period 2 14

Table S7. Key prespecified secondary, exploratory, and post-hoc analyses for Period 1[a] 21

Table S8. Exploratory analysis of percent change of total volume (cm^3) of pre-existing lesions by CT at week 8 and week 28 versus baseline 24

Table S9. Percentages of patients with flare-ups by patient diary and by investigator AE verbatim term in Period 1 (AHO) and Period 2 (mITT) 27

Table S10. Overview of protocol and statistical methodology amendments 28

Table S11. List of key primary and secondary outcomes for Period 1, Period 2, and the open label extension period (per protocol) 38

Figure S1. Activin A activates signaling by FOP-mutant ACVR1 but inhibits signaling by wild type ACVR1 43

Figure S2. Schematic overview of the study design 44

Figure S3. BMP9 levels in patients who experienced TEAEs of epistaxis over time 45

Study sites and investigators

Toronto General Hospital, Toronto, Ontario, Canada: Angela M. Cheung, Lianne Tile

Hôpital Cochin, Service de Rhumatologie, Paris, France: Christian Roux, Jacques Fechtenbaum, Karine Briot, Sami Kolta

Hôpital Lariboisiere, Hospitalier Universitaire Nord, Paris, France: Philippe Orcel, Jeremy Ora, Thomas Funck-Brentano

Department of Pediatrics, Unit of Rare Diseases, IRCCS Istituto Giannina Gaslini, Genoa, Italy: Maja Di Rocco, Annalisa Madeo, Gioacchino A. Rotulo, Marta Bertamino, Sara Signa, Marcello Mariani

Amsterdam UMC, Vrije Universiteit, Amsterdam, The Netherlands: E. Marelise W. Eekhoff, Esmée Botman, Pieter G. Raijmakers, Bernard Smilde, Coen Netelenbos, Sanne Treurniet, Silvia Storoni, Ruben D. De Ruiter

Institute of Medical Sciences, Medical College of Rzeszów University, Rzeszów University, Rzeszów, Podkarpackie, Poland: Arthur Mazur, Jacek Tabarkiewicz, Małgorzata Szczepanek, Katarzyna Wiacek

Department of Rheumatology, Hospital Universitario Ramón y Cajal, Madrid, Spain: Javier Bachiller-Corral, Jesus L. Martos, Monica Vazquez-Diaz, Sandra Garrote-Corral

Centre for Metabolic Bone Disease Royal National Orthopaedic Hospital NHS Trust, London, UK: Richard Keen, Judith S. Bubbear, Vera Choida, Jonathan Moses, Tiara Gill

The Perelman School of Medicine - The University of Pennsylvania, Philadelphia, PA, USA: Mona Al Mukaddam, Frederick S. Kaplan, Staci Kallish, Thomas Guzzo

Department of Medicine, Mayo Clinic, Rochester, MN, USA: Robert J. Pignolo, Lance Mynderse, Matthew Drake, Michael Brown, Stephen Broski, Landon Trost, Timothy Curry, Joshua Savage, Thomas Stewart, Julia Lehman, Alexander Meves

Vanderbilt University Medical Center, Program for Metabolic Bone Disorders Nashville, TN, USA: Kathryn M. Dahir, Daniel Tilden, Kevin Niswender, Laura Bryant

Table S1. TEAEs that occurred in ≥10% of the garetosmab 10 mg/kg Q4W group in the double-blind period of the study (Period 1)[a,b]

**Primary system organ class**<br>**preferred term**	**Placebo**<br>**(*n* = 24)**	**Garetosmab 10 mg/kg Q4W**<br>**(*n* = 20)**
TEAEs, n	246	307
Patients with ≥1 TEAE, n (%)	24 (100)	20 (100)
Skin and subcutaneous tissue disorders, n (%)	11 (45.8)	18 (90.0)
Acne	3 (12.5)	6 (30.0)
Madarosis	0	6 (30.0)
Dry skin	1 (4.2)	3 (15.0)
Pruritus	1 (4.2)	3 (15.0)
Alopecia	1 (4.2)	2 (10.0)
Blister	0	2 (10.0)
Eczema	0	2 (10.0)
Rash	4 (16.7)	2 (10.0)
Rash papular	0	2 (10.0)
Infections and infestations, n (%)	10 (41.7)	14 (70.0)
Rhinitis	0	4 (20.0)
Folliculitis	0	3 (15.0)
Gastroenteritis	1 (4.2)	3 (15.0)
Nasopharyngitis	5 (20.8)	3 (15.0)
Rash pustular	0	2 (10.0)
Abscess limb	0	2 (10.0)
Respiratory, thoracic, and mediastinal disorders, n (%)	9 (37.5)	14 (70.0)
Epistaxis	4 (16.7)	10 (50.0)
Cough	2 (8.3)	3 (15.0)

Gastrointestinal disorders, n (%)	9 (37.5)	13 (65.0)
Diarrhea	6 (25.0)	5 (25.0)
Toothache	0	4 (20.0)
Nausea	2 (8.3)	3 (15.0)
Abdominal pain	1 (4.2)	2 (10.0)
Abdominal pain upper	1 (4.2)	2 (10.0)
Mouth ulceration	1 (4.2)	2 (10.0)
Musculoskeletal and connective tissue disorders, n (%)	20 (83.3)	13 (65.0)
Arthralgia	7 (29.2)	5 (25.0)
Pain in extremity	9 (37.5)	5 (25.0)
Back pain	1 (4.2)	4 (20.0)
Musculoskeletal pain	2 (8.3)	4 (20.0)
Neck pain	3 (12.5)	4 (20.0)
Joint swelling	2 (8.3)	3 (15.0)
Muscle spasms	0	2 (10.0)
Myalgia	3 (12.5)	2 (10.0)
Nervous system disorders, n (%)	11 (45.8)	12 (60.0)
Headache	7 (29.2)	10 (50.0)
Dizziness	2 (8.3)	4 (20.0)
General disorders and administration site conditions, n (%)	9 (37.5)	11 (55.0)
Pyrexia	0	3 (15.0)
Chest discomfort	0	2 (10.0)
Fatigue	2 (8.3)	2 (10.0)
Infusion site pain	0	2 (10.0)
Blood and lymphatic system disorders, n (%)	1 (4.2)	4 (20.0)
Anemia	1 (4.2)	2 (10.0)

[a]Safety analysis set.

[b]A patient with multiple TEAEs is counted once for the same preferred term or system organ class. This table is sorted by descending order of frequency of system organ class and preferred term for the treatment group.

Q4W, every 4 weeks; TEAE, treatment-emergent adverse event.

Table S2. Skin and soft tissue infection TEAEs[a,b]

**Period 1**	**Placebo (*n* = 24)**	**Garetosmab 10 mg/kg Q4W (*n* = 20)**
Skin and subcutaneous infections	3 (12.5)	12 (60)
Acne	3 (12.5)	6 (30.0)
Folliculitis	0	3 (15)
Abscess limb	0	2 (10.0)
Anal abscess	0	1 (5.0)
Anorectal cellulitis	0	1 (5.0)
Pustule	0	1 (5.0)

**Period 2 and open-label extension**	**Total (*N* = 43)**
Skin and subcutaneous infections	29 (67.4)
Acne	16 (37.2)
Abscess limb	7 (16.3)
Anal abscess	5 (11.6)
Folliculitis	4 (9.3)
Furuncle	4 (9.3)
Abscess	3 (7.0)
Subcutaneous abscess	3 (7.0)
Abdominal abscess	2 (4.7)
Groin abscess	2 (4.7)
Perineal abscess	2 (4.7)
Tooth abscess	2 (4.7)
Vulval abscess	2 (4.7)
Abscess oral	1 (2.3)

Abscess rupture	1 (2.3)
Acne cystic	1 (2.3)
Acne pustular	1 (2.3)
Carbuncle	1 (2.3)
Cellulitis	1 (2.3)
Cellulitis orbital	1 (2.3)
Perirectal abscess	1 (2.3)
Pustule	1 (2.3)
Skin infection	1 (2.3)

[a]Safety analysis set.

[b]A patient with multiple TEAEs is counted once for the same preferred term or system organ class. This table is sorted by descending order of frequency of system organ class and preferred term for the treatment group.

Q4W, every 4 weeks; TEAE, treatment-emergent adverse event.

Table S3. SAEs that occurred in the double-blind period of the study (Period 1)[a,b]

Primary System Organ Class<br>Preferred Term	Placebo<br>(n = 24)	Garetosmab 10 mg/kg Q4W<br>(n = 20)
SAEs, n	2	7
Patients with ≥1 SAE, n (%)	2 (8.3)	4 (20.0)
Infections and infestations, n (%)	1 (4.2)	3 (15.0)
Gastroenteritis	0	1 (5.0)
Pneumonia	0	1 (5.0)
Sepsis	0	1 (5.0)
Urinary tract infection	0	1 (5.0)
Gastroenteritis viral	1 (4.2)	0
Gastrointestinal disorders, n (%)	0	1 (5.0)
Intestinal obstruction	0	1 (5.0)
Respiratory, thoracic, and mediastinal disorders, n (%)	0	1 (5.0)
Epistaxis	0	1 (5.0)
Musculoskeletal and connective tissue disorders, n (%)	1 (4.2)	0
Joint swelling	1 (4.2)	0

[a]Safety analysis set.

[b]A patient with multiple SAEs is counted once for the same preferred term or system organ class. This table is sorted by descending order of frequency of system organ class and preferred term for the treatment group.

Q4W, every 4 weeks; SAE, serious adverse event.

Table S4. Bleeding TEAEs

**Period 1**	**Placebo (*n* = 24)**	**Garetosmab 10 mg/kg Q4W (*n* = 20)**
Any bleeding event	9 (37.5)	13 (65.0)
Epistaxis	4 (16.7)	10 (50.0)
Petechiae	0	1 (5.0)
Hemoptysis	0	1 (5.0)
Increased tendency to bruise	0	1 (5.0)
Hematuria	3 (12.5)	0
Metrorrhagia	1 (4.2)	0
Vaginal hemorrhage	1 (4.2)	0
Hematoma	1 (4.2)	0
Chronic pigmented purpura	1 (4.2)	0
Menorrhagia	1 (4.2)	0

**Period 2 and open-label extension**	**Total (*N* = 43)**
Any bleeding event	18 (41.9)
Epistaxis	15 (34.9)
Hematuria	2 (4.7)
Hematochezia	2 (4.7)
Ecchymosis	2 (4.7)
Petechiae	1 (2.3)
Nipple exudate bloody	1 (2.3)
Vaginal hemorrhage	1 (2.3)
Nail bed bleeding	1 (2.3)

Q4W, every 4 weeks; TEAE, treatment-emergent adverse event.

Table S5. SAEs that occurred in the open-label period[a,b]

**Primary system organ class**<br>**preferred term**	**Total**<br>**(*N* = 43)**
SAEs, n	20
Patients with ≥1 SAE, n (%)	13 (30.2)
Infections and infestations, n (%)	7 (16.3)
Abscess	1 (2.3)
Abscess limb	1 (2.3)
Perineal abscess	1 (2.3)
Perirectal abscess	1 (2.3)
Pneumonia	1 (2.3)
Respiratory tract infection viral	1 (2.3)
Subcutaneous abscess	1 (2.3)
General disorders and administration site conditions, n (%)	3 (7.0)
Pyrexia	2 (4.7)
Sudden death	1 (2.3)
Injury, poisoning and procedural complications, n (%)	3 (7.0)
Head injury	1 (2.3)
Skull fracture	1 (2.3)
Splenic rupture	1 (2.3)
Gastrointestinal disorders, n (%)	2 (4.7)
Crohn's disease	1 (2.3)
Intestinal obstruction	1 (2.3)
Nervous system disorders, n (%)	1 (2.3)
Cerebrovascular accident	1 (2.3)
Renal and urinary disorders, n (%)	1 (2.3)

**Primary system organ class preferred term**	**Total (*N* = 43)**
Renal colic	1 (2.3)
Respiratory, thoracic, and mediastinal disorders, n (%)	1 (2.3)
Acute respiratory failure	1 (2.3)

[a]Safety analysis set.

[b]A patient with multiple SAEs is counted once for the same preferred term or system organ class. The table is sorted by descending order of frequency of system organ class and preferred term for the total group. Included are SAEs that were reported on or before the end of study date of October 30, 2020.

SAE, serious adverse event.

Table S6. Summary of all protocol pre-specified secondary and exploratory endpoints for Period 1 and Period 2

A. Period 1 results

Secondary/exploratory objectives	Endpoint	Population	Model[a,b]	Garetosmab	Placebo	Difference (95% CI)	Nominal *P*-value[c]
Secondary, imaging – SUV_{max}	Percent change from baseline in mean SUV_{max} at week 8	AHO	ANCOVA	−22.3 (4.02)	−6.6 (3.70)	–15.7 (−26.8, −4.6)	0.0065
		AHOC	ANCOVA	−22.3 (3.69)	−5.3 (3.54)	−17.0 (−27.4, −6.7)	0.0019
Secondary, imaging - # PET lesions	Change from baseline in number of HO lesions by PET at week 28	AHO	Ranked ANCOVA	−2 (–7, 1)	−2 (−5, 8)	−1 (−2, 0)	0.1768
Secondary, imaging - # PET lesions	Change from baseline in number of HO lesions by PET at week 28	AHOC	Ranked ANCOVA	−2 (–7, 1)	−2 (−5, 8)	−1 (−2, 1)	0.1746
Secondary, imaging - # CT lesions	Change from baseline in number of HO lesions detectable by CT at week 28	AHO	Ranked ANCOVA	0 (−4, 1)	1 (0, 9)	−1 (−1, 0)	0.0192
Secondary, imaging - # CT lesions	Change from baseline in number of HO lesions detectable by CT at week 28	AHOC	Ranked ANCOVA	0 (−4, 1)	1 (0, 9)	−1 (−1, 0)	0.0153
Secondary, P1NP	TWA of percent change from baseline over 28 weeks	AHO	ANCOVA	−25.16 (7.062)	1.01 (6.468)	−26.17 (−45.57, −6.76)	0.0095

		AHOC	ANCOVA	−25.41 (7.023)	3.43 (6.730)	−28.84 (−48.55, −9.13)	0.0052
Secondary, BSAP	TWA of percent change from baseline over 28 weeks	AHO	ANCOVA	−12.36 (4.853)	9.76 (4.456)	−22.12 (−35.38, −8.86)	0.0017
		AHOC	ANCOVA	−12.32 (4.873)	11.08 (4.676)	−23.40 (−37.01, −9.80)	0.0013
Secondary, tAP	TWA of percent change from baseline over 28 weeks	AHO	ANCOVA	−1.77 (5.055)	10.89 (4.649)	−12.66 (−26.51, 1.18)	0.0719
Secondary, tAP	TWA of percent change from baseline over 28 weeks	AHOC	ANCOVA	−1.77 (5.101)	11.12 (4.901)	−12.89 (−27.15, 1.38)	0.0753

78

79 **B. Period 2 Results**

Secondary objectives	Endpoint	Arm	Analysis set	Model	Period 1	Period 2	Comparison	Nominal *P*-value[c]
**Secondary, investigator flare-ups**	Percent of patients with new investigator-assessed flare-ups in Period 2	Placebo/ garetosmab	COVID-19 mITT (n=22)	McNemar	45.5 (10/22) (total # flare ups: 22)	13.6 (3/22) (total # flare ups: 4)	Observed relative risk reduction = 70%	0.0196
		Garetosmab/ garetosmab	COVID-19 mITT (n=18)	Descriptive	11.1 (2/18) (total # flare ups: 2)	5.6 (1/18) (total # flare ups: 1)	-------------------	
**Secondary, patient-reported flare-ups**	Percent of patients with new flare-ups (using patient diary) in Period 2	Placebo/ garetosmab	COVID-19 mITT (n=22)	McNemar	68.2 (15/22) (total # flare ups: 31)	13.6 (3/22) (total # flare ups: 11)	Observed relative risk reduction = 80%	0.0005
		Garetosmab/ garetosmab	COVID-19 mITT (n=18)	Descriptive	33.3 (6/18) (total # flare ups: 12)	22.2 (4/18) (total # flare ups: 6)	-------------------	
**Secondary-Imaging, CT – new relative to week 28**	Number of new HO lesions as assessed by CT only at week 56 relative to week 28 scan	Placebo/ garetosmab	COVID-19 mITT (n=22)	Descriptive + Wilcoxon	Observed rate = 1.27 Total # of lesions = 28	Observed rate = 0.13 Total # of lesions = 3	-------------------	

**Secondary, imaging – CT new relative to baseline**	Number of new HO lesions as assessed by CT at week 56 relative to baseline	Garetosmab/ garetosmab	COVID-19 mITT	Descriptive (bootstrap CI)	Mean: 0.1 (0.0, 0.3)	Mean: 0.1 (0.0, 0.3)	-------------------
**Secondary, imaging – CT new relative to baseline**	Total volume in new HO lesions as assessed by CT at week 56 relative to baseline	Garetosmab/ garetosmab	COVID-19 mITT	Descriptive (bootstrap CI)	Mean: 1.0 (0.0, 2.9)	Mean: 0.4 (0.0, 1.1)	-------------------
**Secondary, imaging – CT new relative to baseline**	Percent of patients with new HO lesions as assessed by CT at week 56 relative to baseline	Garetosmab/ garetosmab	COVID-19 mITT	Descriptive	11.1 (2/18)	11.1 (2/18)	-------------------
**Secondary, imaging – PET new relative to baseline**	Number of new lesions as assessed by 18F-NaF PET at week 56 relative to baseline	Garetosmab/ garetosmab	COVID-19 mITT	Descriptive (bootstrap CI)	Mean: 0.1 (0.0, 0.2)	Mean: 0.1 (0.0, 0.2)	-------------------
**Secondary, imaging – PET new relative to baseline**	Total lesion activity in new HO lesions as assessed by 18F-NaF PET at week 56 relative to baseline	Garetosmab/ garetosmab	COVID-19 mITT	Descriptive (bootstrap CI)	4.7 (0.0, 12.7)	0.7 (0.0, 1.8)	-------------------
**Secondary, imaging – PET**	Percent of patients with new	Garetosmab/ garetosmab	COVID-19 mITT	Descriptive	5.6 (1/18)	5.6 (1/18)	-------------------

**new relative to baseline**	HO lesions as assessed by 18F-NaF PET at week 56 relative to baseline						
**Secondary, PET – SUV_{max}**	Percent change from week 28 in SUV_{max} to week 56	Placebo/ garetosmab	COVID-19 mITT	Bootstrap + Wilcoxon	−16.8 (−24.6, −8.7)	−30.3 (−36.6, −24.1)	−13.8 (−24.6, −2.6)
		Garetosmab/ garetosmab	COVID-19 mITT	Bootstrap	−32.1 (−41.7, −22.9)	−16.6 (−29.0, −6.5)	15.8 (3.0, 27.5)
**Secondary, PET – SUV_{max}**	Percent change from baseline in SUV_{max} to week 56	Garetosmab/ garetosmab	COVID-19 mITT	Descriptive	−32.1 (−41.7, −22.9)	−41.9 (−55.1, −28.8)	-------------------
**Secondary, PET – TLA**	Percent change from week 28 in TLA by ^{18}F-NaF PET to week 56	Placebo/ garetosmab	COVID-19 mITT	Bootstrap + Wilcoxon	48.2 (0.4, 120.2)	−16.4 (−30.6, −2.5)	−66.9 (−154.6, −6.4)
		Garetosmab/ garetosmab	COVID-19 mITT	Bootstrap	−15.9 (−34.5, 3.5)	−3.6 (−16.8, 7.9)	14.0 (−4.1, 33.7)
**Secondary, PET – TLA**	Percent change from baseline in TLA by ^{18}F-NaF PET to week 56	Garetosmab/ garetosmab	COVID-19 mITT	Bootstrap	−15.9 (−34.5, 3.5)	−16.4 (−39.8, 8.8)	-------------------
**Secondary, CT – total volume**	Percent change from week 28 in the total volume of HO lesions as assessed by CT to week 56	Placebo/ garetosmab	COVID-19 mITT	Bootstrap + Wilcoxon	34.0 (1.7, 91.9)	4.5 (−1.7, 11.7)	−29.5 (−91.2, 7.0)
		Garetosmab/ garetosmab	COVID-19 mITT	Bootstrap	7.5 (2.0, 13.8)	−3.5 (−9.6, 1.4)	−11.1 (−22.8, −1.3)

**Secondary, CT – total volume**	Percent change from baseline in the total volume of HO lesions as assessed by CT to week 56	Garetosmab/ garetosmab	COVID-19 mITT	Bootstrap	7.5 (2.0, 13.8)	2.6 (−2.0, 7.1)	-------------------
**Secondary, PET – number of lesions**	Change from week 28 in number of HO lesions by ^{18}F-NaF PET to week 56	Placebo/ garetosmab	COVID-19 mITT	Bootstrap + Wilcoxon	−1.0 (−2.0, 0.1)	−2.1 (−2.9, −1.4)	−1.1 (−3.0, 0.4)
		Garetosmab/ garetosmab	COVID-19 mITT	Bootstrap	−2.4 (−3.5, -1.4)	−1.4 (−2.1, −0.7)	1.3 (−0.2, 2.8)
**Secondary, PET – number of lesions**	Change from baseline in number of HO lesions by ^{18}F-NaF PET at week 56	Garetosmab/ garetosmab	COVID-19 mITT	Bootstrap	−2.4 (−3.5, −1.4)	−4.0 (−4.9, −3.2)	-------------------
**Secondary, CT – number of lesions**	Change from week 28 in number of HO lesions by CT to week 56	Placebo/ garetosmab	COVID-19 mITT	Bootstrap + Wilcoxon	1.1 (0.5, 2.0)	−0.2 (−0.5, 0.1)	−1.3 (−2.4, −0.5)
		Garetosmab/ garetosmab	COVID-19 mITT	Bootstrap	−0.4 (−1.1, 0.2)	−0.1 (−0.2, 0.0)	0.3 (−0.2, 0.9)
**Secondary, CT – number of lesions**	Change from baseline in number of HO lesions by CT to week 56	Garetosmab/ garetosmab	COVID-19 mITT	Bootstrap	−0.4 (−1.1, 0.2)	−0.4 (−1.2, 0.2)	-------------------
**Secondary, NRS**	Daily pain due to FOP as measured using the daily NRS	Placebo/ garetosmab	COVID-19 mITT	Bootstrap + Wilcoxon	1.79 (1.0, 2.7)	1.60 (0.9, 2.4)	−0.19 (−0.6, 0.1)
		Garetosmab/ garetosmab	COVID-19 mITT	Bootstrap	1.31 (0.7, 2.1)	1.15 (0.5, 2.0)	−0.16 (−0.4, 0.1)

**Secondary, glucocorticoids**	Total dosage of glucocorticoid use over time	Placebo/ garetosmab	COVID-19 mITT	Bootstrap + Wilcoxon	4186.4 (2198.6, 6451.8)	1099.1 (411.6, 1925.0)	−3087.3 (−5514.1, −982.3)
		Garetosmab/ garetosmab	COVID-19 mITT	Bootstrap	691.7 (222.2, 1260.3)	493.9 (47.5, 1080.6)	−197.8 (−2230, 2000)

All the McNemar test's that were performed had at least 10 discordant pairs for accurate computation of the Chi-squared test statistic.

[a]For ANCOVA and MMRM, LS mean (SE) and LS mean differences (95% CI) are shown. For Fisher's exact test, numbers and proportions of patients are shown.

[b]For ranked ANCOVA, median (mix, max) within-group and median differences are shown. For ranked ANCOVA model, median treatment difference and 95% CI was derived from the Hodges-Lehmann estimate and Moses distribution-free CI, respectively. *P*-value was estimated from ANCOVA on ranked responses.

[c]*P*-values are given for descriptive purposes to evaluate nominal strength.

^{18}F-NaF, fluorine-18-labelled sodium fluoride; AHO, patients with at least one active heterotopic ossification lesion at baseline; AHOC, patients with at least one active heterotopic ossification lesion at baseline and with classic $ACVR1^{R206H}$ mutation; ANCOVA, analysis of covariance; BSAP, bone specific alkaline phosphatase; CAJIS, Cumulative Analog Joint Involvement Scale; CI, confidence interval; CRP, C-reactive protein; CT, computerized tomography; EQ5D, health status questionnaire by EuroQol Group; FOP, fibrodysplasia ossificans progressiva; GEE, general estimating equation; HO, heterotopic ossification; IADL, independent activity daily living questionnaire; LS, least-squares; mITT, modified intention to treat; MMRM, mixed model with repeated measures; NRS, numeric rating scale; PET, positron emission tomography; P1NP, procollagen type 1 N-Terminal propeptide; SE, standard error; SUV_{max}, maximum standardized uptake value; tAP, total alkaline phosphatase; TLA, total lesion activity; TWA, time-weighted average.

Table S7. Key prespecified secondary, exploratory, and post-hoc analyses for Period 1[a]

Assessment (type)	Outcome	Garetosmab	Placebo	Reduction	*P*-value[b]
New ^{18}F-NaF PET lesions (post-hoc)	Total lesion activity in new lesions per patient by ^{18}F-NaF PET at week 28 (mean)[c]	5.22 g	205.99 g	97% relative reduction	0.009
New CT lesions (post-hoc)	Total volume of new lesions per patient by CT at week 28 (mean)[c]	1.06 cm^3	10.21 cm^3	90% relative reduction	0.017
^{18}F-NaF PET lesions (prespecified exploratory)	Percent of patients with new bone lesions measured by ^{18}F-NaF PET over 28 weeks[d]	15.0 (3/20)	45.8 (11/24)	67.4% reduction in risk (relative risk=0.33)	0.05
New CT lesions (post-hoc)	Percent of patients with new bone lesions measured by CT over 28 weeks[d]	15.0 (3/20)	45.8 (11/24)	67.4% reduction in risk (relative risk=0.33)	0.05
New ^{18}F-NaF PET lesions (post-hoc)	Number of new bone lesions measured by ^{18}F-NaF PET over 28 weeks[e]	Rate: 0.15<br>Count: 3<br>LA/pt: 34.8 g[f]	Rate: 1.2<br>Count: 29<br>LA/pt: 449.4 g[f]	87.4% decrease (0.13 rate ratio)	0.006
New CT lesions (post-hoc)	Number of new bone lesions per patient measured by CT over 28 weeks[e]	Rate: 0.15<br>Count: 3<br>Vol/pt: 7.1 cm^3[f]	Rate: 1.1<br>Count: 27<br>Vol/pt: 22.3 cm^3[f]	86.7% decrease (0.13 rate ratio)	0.009

^{18}F-NaF PET lesions (prespecified secondary)	Number of HO lesions (95% CI) by ^{18}F-NaF PET per scan over 28 weeks[e]	3.5 (2.8, 4.3)	4.8 (4.0, 5.7)	27% decrease (0.73 rate ratio)	0.03
Imaging – SUV_{max} (prespecified secondary)	Percent change from baseline in SUV_{max} at week 8, LS mean (SE)[g]	−22.3 (4.0)	−6.6 (3.7)	−15.7 (−26.8, −4.6)	0.007
Imaging – SUV_{max} (post-hoc)	Percent change from baseline in SUV_{max} at week 28, LS mean (SE)[g]	−34.0 (3.7)	−11.6 (3.4)	−22.4 (−32.6, −12.2)	<0.001
Imaging – number of CT lesions (prespecified secondary)	Number of HO lesion/scan (95% CI) through 28 weeks[e]	5.3 (4.7, 6.1)	6.3 (5.6, 7.0)	16% decrease (0.84 rate ratio)	0.07

The total lesion activity is derived from the sum of the products between the mean standardized uptake value in units of g/cm^3 and the volume in cm^3 of each pre-existing and new lesion, which results in units of g.

[a]Active HO analysis set.

[b]Nominal *P*-value.

[c]Wilcoxon test.

[d]Fisher's exact test.

[e]Negative binomial model.

[f]LA/pt, Vol/pt: lesion activity per patient and volume per patient are calculated by averaging new lesion activities/volume among patients who had new lesions.

[g]ANCOVA.

^{18}F-NaF, fluorine-18-labelled sodium fluoride; ANCOVA, analysis of covariance; CI, confidence interval; CT, computed tomography; HO, heterotopic ossification; LS, least squares; PET, positron emission tomography; Q4W, every 4 weeks; SUV_{max}, maximal standardized uptake value; SE, standard error.

Table S8. Exploratory analysis of percent change of total volume (cm^3) of pre-existing lesions by CT at week 8 and week 28 versus baseline

	**Placebo (*n* = 24)**	**Garetosmab 10 mg/kg Q4W (*n* = 20)**
**Baseline**		
Patients included, n	24	20
Total pre-existing lesions,[a] n	134	119
Total volume of pre-existing HO lesions, cm^3		
n	24	20
Mean (SD)	235.8 (253.3)	251.4 (327.9)
Median	159.7	112.3
Q1 : Q3	113.6 : 267.3	46.5 : 242.9
Min : Max	17.3 : 1173.3	5.2 : 1046.6
**Week 8**		
Patients included, n	24	20
Total pre-existing lesions, n	136	114
Total volume of pre-existing HO lesions, cm^3		
n	24	20
Mean (SD)	245 (264.8)	258.3 (330.3)
Median	174.9	112
Q1 : Q3	120.5 : 261.3	54.8 : 258.7
Min : Max	28:57.8	7.8 : 1076.8
Percent change from baseline		
n	24	20
Mean (SD)	-0.2 (18.0)	11.2 (18.1)
Median	1.4	5.6

Q1 : Q3	-2.8 : 8.1	-0.3 : 12.4
Min : Max	-55.1 : 37.7	-7.8 : 59.1
Median % difference[b], week 8 vs. baseline (95% CI)	-1.61 (6.37, 3.79)	-6.86 (-19.7, -1.93)
Nominal *P*-value vs. baseline	0.509	0.006
Adjusted nominal *P*-value vs. baseline	0.509	0.024
**Week 28**		
Patients included, n	24	20
Total pre-existing lesions, n	136	110
Total volume of pre-existing HO lesions, cm^3		
n	24	20
Mean (SD)	242.2 (244.5)	256.9 (332.5)
Median	194.1	116.4
Q1 : Q3	117.6 : 270.9	53.6 : 264.8
Min : Max	15.3 : 1147	6.9 : 1077.2
Percent change from baseline		
n	24	20
Mean (SD)	11.1 (40.7)	6.4 (13.4)
Median	1.0	4.0
Q1 : Q3	-1.2 : 8.1	-2.8 : 12.4
Min : Max	-36.0 : 189.0	-12.7 : 40.6
Median % difference[b], week 28 vs. baseline (95% CI)	-2.65 (-8.44, 0.59)	-4.75 (-12.1, 0.33)
Nominal *P*-value vs. baseline	0.128	0.070
Adjusted nominal *P* value vs. baseline	0.256	0.210

[a]Pre-existing lesions refers to pre-existing HO lesions. [b]Median difference from baseline and 95% CI was derived from the Hodges-Lehmann estimate and Moses distribution-free CI, respectively. The *P*-value was obtained from a paired Wilcoxon signed rank test to compare the percent change of total volume of pre-existing lesions at week 8 and week 28 versus baseline.

The adjusted *P*-value accounts for multiple comparisons using the Holm method.

CI, confidence interval; CT, computed tomography; HO, heterotopic ossification; Q4W, every 4 weeks; SD, standard deviation.

Table S9. Percentages of patients with flare-ups by patient diary and by investigator AE verbatim term in Period 1 (AHO) and Period 2 (mITT)

Incidence of flare-up events, *n* (%)

Double-blind Period 1, inter-group comparison

	Placebo (*n* = 24)	Garetosmab (*n* = 20)	*P*-value
Patient diary	17 (70.8)	7 (35.0)	0.03
Investigator AE verbatim term	10 (41.7)	2 (10.0)	0.04

Period 2 compared to Period 1, intra-group comparison

	Placebo (Period 1) vs. garetosmab (Period 2) (*n* = 22)[a,b]		*P*-value
Patient diary	15 (68.2)	3 (13.6)	0.0005
Investigator AE verbatim term	10 (45.5)	3 (13.6)	0.019

[a]No comparison performed since the intent is to evaluate persistency of garetosmab effect.

[b]Of the 24 patients on placebo in Period 1, 22 went on to garetosmab in Period 2. Shown here are only those 22 patients who transitioned from placebo to garetosmab.

AE, adverse event; AHO, active heterotopic ossification analysis set; HO, heterotopic ossification; mITT, modified intent-to-treat.

Table S10. Overview of protocol and statistical methodology amendments

	**Amendments to statistical methodology**
1.	In January 2019, an urgent safety measure was implemented due to a serious event of epistaxis (resulting in hospitalization; Section 5.2.1.5) and the totality of data regarding the occurrence of epistaxis in the ongoing study at that time. This resulted in the implementation of Protocol Amendment 3 (Appendix 16.1.1), which included the following updates:<br>• Inclusion of new exclusion criteria for patients on concomitant antiplatelet therapy, history of severe non-traumatic bleeding, and history of bleeding diathesis (Section 3.3.1).<br>• Inclusion of moderate to severe episodes of non-traumatic bleeding and moderate to severe epistaxis as AESIs; Section 5.2.1.7).<br>• The use of anti-inflammatory drugs that inhibited platelet function or were otherwise associated with an increased risk of bleeding was discouraged to the extent possible.<br>• The low dose of acetylsalicylic acid was clarified (≤100 mg/day).<br>• Baseline and post-treatment laboratory measures of coagulation parameters and platelet effector function were added.<br>• A baseline exploratory research sample was added to explore mechanism of epistaxis and garetosmab mechanism of action.<br>• Antiplatelet therapy, anticoagulants, and herbal supplements (that inhibit platelet function or are associated with increased risk of bleeding) were added to the list of prohibited medications.
2.	In October 2020, a second urgent safety measure was issued to halt garetosmab dosing after a fatal SAE that occurred during treatment. Preliminary findings from a macroscopic post-mortem examination suggested gross hemorrhage in the right lung. However, upon definitive final review by two pathologists, the final cause of death was considered sudden cardiac arrest, with additional findings of extensive granulomatous inflammation most likely attributable to chronic ongoing aspiration of foreign material (Section 5.2.1.4). Four other deaths had been previously reported during the open-label period of the study (Section 5.2.1.4). Before garetosmab dosing was halted, all subjects had completed Period 3 (i.e., reached 76 weeks) and all efficacy assessments had been completed. In September 2021, the sponsor decided to close the study (final cutoff, Section 1.4), after all participants had performed all efficacy and other relevant assessments described in the study protocol and open-label administration of garetosmab was not planned to be reinitiated.
3.	By the time COVID-19 was declared as a global pandemic by the World Health Organization (March 11, 2020), all patients had completed Period 1 and most patients had completed Period 2 of the study. The sponsor decided to continue the study based on the assessment that it could be conducted without jeopardizing patient safety, data integrity, or compliance with relevant Regulatory Guidance.<br>Because of the public health emergency related to the COVID-19 pandemic, study procedures were adapted, and are reflected in Appendix 16.1.1, Protocol Amendment 6. Study procedures, including study drug administration, were allowed at alternative sites, which were established to minimize study patient(s)' exposure to the SARS-CoV-2 virus. It was acceptable to perform the study visit 18 (week 56) and/or visit 23 (week 76) imaging exams employing only whole-body low-dose CT scans because of potential unavailability of the ^{18}F-NaF tracer necessary for the PET scan, though this was not ultimately needed. In addition, the time window for study visit 18 (week 56) was made flexible, as long as any changes in the schedule were documented. A new

	dataset was also defined in Protocol Amendment 6 to mitigate the effects of the public health emergency on study outcomes, applicable to Period 2 and Period 3 (COVID-19 mITT, see Section 3.7.2.1).<br><br>Formal documentation of deviations to standard operating procedures or other controlled procedural documents is in Appendix 16.1.18.<br><br>An assessment of protocol deviation trending during the pandemic is in Appendix 16.1.18.<br><br>All temporary mechanisms used and deviations from planned study procedures were documented as being related to COVID-19.
4.	Upon Protocol Amendment 1, the scope of patient eligibility was expanded by opening enrollment to FOP patients with any *ACVR1* mutation known to cause the disease, not only the classic *ACVR1*^{R206H} mutation. This expansion was based on in vitro and in vivo studies that demonstrated that all the FOP-causing mutations in *ACVR1* render this receptor responsive to activin A, much like the prototypical *ACVR1*^{R206H} variant (see Section 1.2). These results indicated that garetosmab is likely to be effective in blocking HO irrespective of the specific FOP-causing variants of *ACVR1* present in any given patient (Appendix 16.1.1, Protocol Amendment 1). To support the analyses of study results, the *ACVR1* genotype initially documented by investigators at enrollment was confirmed retrospectively in a central laboratory for all patients. Efficacy of garetosmab was assessed irrespective of ACVR1 mutation and separately for the patients with an *ACVR1*^{R206H} mutation.
5.	Observations from Period 1 led to a redefinition of pre-specified hypotheses for Period 2. In Period 1, non-statistically significant reductions were observed in both HO activity (as measured by ^{18}F-NaF PET) and in HO volume (as measured by CT) (Section 5.1.1.1). Further examination of these data revealed that changes in these measures were entirely driven by activity and growth associated with new HO lesions. These observations led to the redefinition of endpoints for Period 2 with focus upon the growth and activity of new HO lesions. The pre-defined study hypothesis for Period 2 was amended in Protocol Amendment 6 (Appendix 16.1.1) as follows:<br>• Treatment with garetosmab prevents the formation of new HO lesions in patients with FOP.<br>• This hypothesis was tested primarily in the group of patients who crossed over from placebo to garetosmab in Period 2. Analysis in patients who were treated with garetosmab in both Period 1 and Period 2 aimed to provide insight into the durability of pharmacological effect.
6.	No formal hypotheses were formulated for Period 3. The primary goals of Period 3 were to assess the persistence of efficacy and to assess the long-term safety of garetosmab.

**Protocol Amendment 6**	
**Change**	**Rationale for change**
Added statements to address the impact of the COVID-19 pandemic:<br>• That temporary, alternative mechanisms and flexibility in the visit schedule that may be used to ensure the continuity of clinical study conduct and oversight in light of the public health emergency related to COVID-19.<br>• Defined the COVID-19 mITT principle that will be used for the week 56 primary analysis of efficacy endpoints related to imaging	To explain the plan for ensuring continuity of clinical study activities and study oversight activities during the COVID-19 public health emergency.<br><br>To address the potential impact of the COVID-19 pandemic on the collection of imaging scans and/or missed or delayed study drug administration

Added a summary of the primary analysis results for efficacy and safety in Period 1 (week 28)<br>Added a summary of risks for epistaxis and skin and soft tissue infection	To provide a high-level summary of the study results for efficacy and safety for Period 1 (week 28)
Added a new hypothesis for Period 2 (week 56; open-label treatment period) based on the current understanding of the biology of FOP	To update the planned efficacy analysis for Period 2 (week 56; open-label treatment period) based on the primary efficacy analysis results from Period 1 (week 28; double-blind, placebo-controlled period)
Added a rationale for the analyses to confirm the new hypothesis (week 56) with additional alpha of 0.1 specified for control for Type I multiplicity for Period 2 (week 56).<br>Revised the description of the clinical endpoint measures	To specify new analyses which can handle the possibility of patients with missing week 56 imaging assessment (due to COVID-19 disruption for the study) and are consistent with regulatory guidance for analysis for rare disease patient populations.
Added new endpoints for Period 2 (week 56) to confirm the understanding of the biology of new HO bone lesions:<br>• Primary efficacy endpoint<br>• Key secondary efficacy endpoints<br>• Other secondary efficacy endpoints	To describe the added endpoints or analysis in Period 2 (open label treatment period; week 56)
Added a new primary endpoint (week 56) based on low-dose CT assessment<br>Reorganized the study variables section to present endpoints by Period 1 (week 28), Period 2 (week 56), and Period 3 (week 76)	To specify a primary endpoint using an imaging assessment that may be easier to perform than the PET assessment during the COVID-19
Added description of the new statistical analyses for efficacy in Period 2 (week 56)<br>Updated the description of the analysis of efficacy endpoints in Period 1 (week 28) was updated (Amendment 6) to match the final statistical analysis plan for Period 1<br>Clarified the description of drug (functional garetosmab) and pre-existing (total activin A) assessed	To specify the new statistical analyses in Period 2 (open-label treatment period; week 56) based on the primary efficacy analysis results from Period 1 (week 28; double-blind, placebo-controlled period)<br>To be consistent with the final statistical analysis plan for Period 1 (double-blind treatment period; week 28) issued before the database lock<br>To be consistent with the drug and pre-existing assays
Added a description of the criteria for continued access to garetosmab for patients who completed Period 3 (week 76)	To be consistent with our corporate policy governing access to investigational drugs in confirmatory clinical studies.

Removed the requirement for a safety discussion with investigators prior to each administration of study drug after week 56. Therefore, after week 56 the need for such interactions will be decided on a case-by-case basis.<br><br>Reduced the frequency of blood sample collection to every 3 months after week 56 for assessments of safety and ADAs	The safety results for Period 1 (week 28) have not identified significant safety concerns that require continued safety discussions or frequent safety laboratory assessments.<br><br>The reduction in assessments for safety and ADAs after week 56 reduces the burden on the patient.
Added assessments for FOP I-ADL, EQ-5D-3L, CAJIS and spirometry and collection of blood samples for measurement of drug and pre-existing, and ADAs every 24 weeks after week 76.	To support assessment of safety, PK, pre-existing, and ADAs.
Will ask patients who permanently discontinue study treatment but do not withdraw from the study to return to the clinic for blood sample collection for measurement of serum drug and pre-existing concentration, at 4 weeks, 8 weeks, and 16 weeks, and 28 weeks after the patient's last dose.<br><br>Assessment of ADAs at the patient's last visit.	The request will support assessment of:<br>• Drug elimination phase<br>• Safety for follow-up of patients<br>• Potential disease rebound<br>• ADAs at the end of the study
Updated 'Pharmacovigilance and Risk Management' to 'Global Patient Safety' in various sections	Text change reflects the new organization name
**Protocol Amendment 5**	
**Change**	**Rationale for change**
The option for SC administration is removed as the concentration of the current formulation is not suitable for SC use.	The purpose of this amendment is to revise the description of study treatment based on a new liquid formulation.
**Protocol amendment 4**	
**Change**	**Rationale for change**
The order of statistical testing of the primary efficacy and key secondary endpoints is: Baseline active HO analysis set first, and baseline active HO classic *ACVR1*^{R206H} mutation analysis set second.	This is a non-substantial amendment to change the hierarchy order of statistical testing for the primary efficacy and key secondary endpoints in response to comments from a health authority.
**Protocol Amendment 3**	
**Change**	**Rationale for change**

In order to mitigate the potential risk for epistaxis, three exclusion criteria were added:<br>• #18 Patients who are on concomitant antiplatelet therapy (e.g., clopidogrel), anti-coagulants (e.g., warfarin, heparin, factor Xa inhibitor, or thrombin inhibitors) in the last 30 days or within five half-lives of the therapy, whichever is longer. Low-dose acetylsalicylic acid (aspirin) is acceptable.<br>• #19 Patients with a history of severe, non-traumatic bleeding requiring transfusion or hospitalization for hemodynamic compromise<br>• #20 Patients with a known pre-existing medical history of a bleeding diathesis (e.g., hemophilia A, von Willebrand's Factor deficiency, platelet count ≤$20x10^9$/L).<br>As an additional mitigation strategy, investigators should discourage the use of anti-inflammatory drugs that inhibit platelet function or are otherwise associated with an increased risk of bleeding, to the extent possible.<br>Clarified the low dose acetylsalicylic acid (aspirin; [≤100 mg/day])	The purpose of this amendment is to revise the protocol to reflect new safety information relating to a potential risk for epistaxis.
Added baseline exploratory research sample to explore mechanism of epistaxis and garetosmab mechanism of action<br>Added antiplatelet therapy, anticoagulants, and herbal supplements (that inhibit platelet function or are associated with increased risk of bleeding) to the list of prohibited medications<br>To increase early detection of epistaxis or bleeding events, the following were added as AESIs:<br>• Moderate-to-severe episodes of non-traumatic bleeding<br>• Moderate epistaxis (defined as any episode lasting longer than 30 minutes or requiring professional medical intervention)<br>• Severe epistaxis (based on definition of an SAE as per protocol)	To aid in the exclusion of patients who may have existing propensity for bleeding, and to assess the effect of garetosmab administration, baseline and post-treatment laboratory measures of coagulation parameters and platelet effector function were added.
**Protocol Amendment 2**	
**Change**	**Rationale for change**

To support interpretation of the study results, all patients will have their *ACVR1* gene sequenced during the study (mandatory).<br><br>The primary statistical analyses for the study will be based on the original study design, not the expanded study population.	The purpose of this amendment is to remove the requirement that patients have the specific classic *ACVR1*^{R206H} mutation. This change is based on in vitro studies which demonstrated that different mutations in ACVR1 receptors transduced bone morphogenic protein signaling when stimulated with activin A. These results indicate that garetosmab may be effective for all FOP mutations. This change expands the scope of the study to include patients with a clinical diagnosis of FOP who may have different *ACVR1* mutations.
Use a hierarchical testing procedure instead of the Hochberg procedure. Revised statistical plan:<br>• Specify hierarchical testing of the four efficacy primary endpoints<br>• Specify that the baseline active HO analysis set includes all randomized patients who had active HO lesion at baseline<br>• Specify that patients will be randomized to garetosmab or placebo in a 1:1 ratio by *ACVR1* mutation (classic *ACVR1*^{R206H}, other) in addition to gender and presence/absence of baseline active HO lesions.<br>• Specify that results are analyzed first in patients with the *ACVR1*^{R206H} mutation and secondly in patients with any ACVR1 mutation.<br>• Make minor corrections to descriptions and tables	The statistical analysis was revised to address feedback from regulatory agencies, as well as addressing the changes related to inclusion of patients with *ACVR1* mutations.
The duration of time for patients to maintain highly effective contraception was extended to 30 weeks after the last dose of study drug to address feedback from a regulatory agency.<br><br>Added an exclusion criterion “Previous history or diagnosis of cancer” and updated the SAE definition to note that “a new diagnosis or progression of a malignancy in patients enrolled in the study will also be considered an SAE” to address feedback from a regulatory agency.<br><br>Deleted ‘futility’ as a reason for premature termination of the study<br><br>Added individual-level dose-modification criteria, clarified the study-level dose modification, and specified that a pregnancy will be tracked until delivery along with a 3-month postnatal follow-up period for the infant, in response to a health authority request.	To address feedback from a regulatory agency:

Added the week 76 time point to the additional analyses of safety and efficacy data to be performed following the open-label treatment period, for consistency with the planned analyses.	
Added a secondary objective and secondary endpoints for assessment of the effect of garetosmab, between week 28 and week 56, on the number, activity, and volume of HO lesions identified by ^{18}F-NaF PET or by CT in patients who switch from placebo to garetosmab at week 28.	
Added assessment of hs-CRP as a biomarker<br>Added assessment of magnesium to the clinical chemistry panel<br>Added an endpoint for FEV1 of spirometry<br>Added measurement of height at baseline	
Provided more detail about the frequency of IDMC meetings and clarified the monitoring for potential AEs in male patients.	
Clarified the rationale for use of the SC route of administration.<br>Provided details on how the safety of each dose will be reviewed prior to next dose.	
Clarified the PET/CT procedures and the exploratory endpoints assessed by PET/CT.	
Clarified that treatment with imatinib or isotretinoin is also exclusionary and are prohibited medications	
Added or updated footnotes to indicate:<br>• That patients can be rescreened for study participation<br>• That measurement of a patient's height may not be precise or not possible to be assessed<br>• Clarification of the urinalysis procedure	Provide more detail about the clinical endpoint measures and study assessments
Removed futility as a reason for study termination	
Minor edits for clarification of study terminology and correction:<br>• Clarified the description of the sample size<br>• Corrected an error in description of the full analysis set in the synopsis	

Minor edits for clarification of study terminology, corrections, and minor changes:<br>• Period 1, Period 2, and Period 3 instead of "treatment period 1", "treatment period 2", and "treatment period 3"<br>• "Diary" has been replaced with "e-diary".<br>• "FOP disease diary" has been replaced with "FOP disease activity diary".<br>• "Menstrual History" instead of "Menstrual Questionnaire"<br>• "FOP Independent Activities of Daily Living" instead of "Activity of Daily Living"<br>• "FCV" to "FVC"<br>• Subjects changed to patients<br>• Adding "Doppler" to scrotum ultrasound	
**Protocol Amendment 1**	
**Change**	**Rationale for change**
Changed "the 24-week follow-up period" to the "follow-up treatment period" (Period 3). Each patient will continue to receive garetosmab every 4 weeks during this period until they complete the week 76 visit, and all data have been collected and validated through the time when the last patient randomized into the study completes the week 28 visit (Treatment Period 1), and results of the primary analyses of safety and efficacy are available to the sponsor. If at the time patients reach the week 76 visit data from the last patient randomized into the study through the week 28 visit have not been collected and validated, and results of the primary analyses of safety and efficacy are not yet available to the sponsor, patients will continue to receive garetosmab every 4 weeks, beyond week 76.<br>Removed footnote #17 from Schedule of Events, Table 3, as this footnote addressed the transition from on-treatment to off-treatment which is no longer relevant to the study.<br>The deletion of this footnote caused footnote #19 for Daily Pain NRS and Daily FOP disease activity diary from Schedule of Events, Table 1 to be changed to footnote#18.<br>Removed the post-treatment period as the third observation period, because treatment with garetosmab will continue during treatment follow-up (Period 3) through to the end of the study.	Treatment with garetosmab will continue provided that no safety signals are identified during continuous monitoring of the study by the investigator, medical monitor and IDMC. In addition, all patients will be required to maintain contraception during this period.
"End of Treatment" was removed from visit 17 of Period 2 (open label treatment period), as the end of study will occur after Period 3 (follow-up treatment period).	

Schedule of Events Table 3: Addition of a new column entitled: “End of Study/Week 80” to denote the new end of the study and to indicate the procedures that will be conducted during this period in the event that data from the last patient randomized into the study through the week 28 visit (Treatment Period 1) have not been collected and validated, and results of the primary analyses of safety and efficacy are not yet available to the sponsor.<br>A new row entitled “Administer garetosmab” was added.	Reduced visit windows in Period 3 from ±14 days to ±7 days, as patients will continue to be treated with garetosmab during this period.
Removed the sentence regarding patients entering the off-drug treatment phase after early termination as the off-drug phase was removed and the statement is no longer applicable.	
Clarified that PET/CT scans will be read and analyzed using a blinded central reading center during the study; however, at baseline, added the provision that the investigator will be allowed to independently read and analyze PET/CT images. Baseline PET/CT images will be performed before each patient is randomized into the study. As these baseline images do not contain any unblinding data, the principal investigator may utilize these images as part of standard of care, resulting in not having to duplicate the imaging assessments and thereby reducing the patients’ total annual radiation exposure.	
Added the requirement that any incidental findings identified through PET/CT scans will be reported to the investigator and medical monitor, and that the investigator will communicate any findings to the patient.<br>Added the requirement that any incidental findings identified through the optional DNA analysis will be reported to the investigator and/or the Vanderbilt University Medical Center (VuMC) Unsolicited Finding Committee for appropriate follow-up with the patient. Also clarified that, by signing the informed consent for the optional DNA sub-study, the patient agrees to be informed of any incidental findings.	
Added whole body ^{18}F-NaF PET and whole-body low-dose CT imaging at week 76 (day 533 [± 7 days]) for a total of five scans during the study. This will allow for the evaluation of longer-term effects of garetosmab (i.e., an additional 20 weeks of treatment) in patients with FOP. As a result of the additional scan, the total effective dose range approximation was changed from 21 mSv to 25 mSv.	

Added that ECG and symptom-directed physical examination may be performed during the follow-up treatment period (Period 3, after week 56) with garetosmab at the investigator’s discretion, and that urine pregnancy testing will be performed prior to each garetosmab dose administration, and a serum pregnancy test will be performed at the patient’s end of study visit. PK/ADA sampling will continue to be performed at all visits after week 56.	
Removed the post-treatment period from the definitions of AEs, as the post-treatment period is no longer relevant.	
Added the week 76 time point to the additional analyses of safety and efficacy data to be performed following the open-label treatment period, for consistency with the planned analyses in the global study.	

Period 3 was the open-label extension portion of the trial that extended beyond 56 weeks.

^{18}F-NaF, ^{18}F-sodium fluoride; ADA, antidrug antibody; AE, adverse event; AESI, adverse event of special interest; CAJIS, Cumulative Analog Joint Involvement Scale; COVID-19, coronavirus disease 2019; CT, computed tomography; ECG, electrocardiogram; EQ-5D-3L, EuroQol 5 dimensions questionnaire with a 3-level scale; FEV1, forced expiratory volume in 1 second; FOP I-ADL, fibrodysplasia ossificans progressiva Independent Activities of Daily Living; FVC, forced vital capacity; hs-CRP, high-sensitivity C-reactive protein; IDMC, independent data monitoring committee; mITT, modified intent-to-treat; NRS, numeric rating scale; PET, positron emission tomography; PK, pharmacokinetics; SAE, serious adverse event; SARS-CoV-2, severe acute respiratory syndrome coronavirus 2; SC, subcutaneous.

Table S11. List of key primary and secondary outcomes for Period 1, Period 2, and the open label extension period (per protocol)

**Period 1[a]**
Primary safety endpoint
Incidence and severity of TEAEs through the end of the Period 1 (baseline to week 28)
Primary outcome measures
[1] TWA (standardized AUC) of the percent change in TLA by ^{18}F-NaF PET using AHO (baseline to week 28)
[2] Percent change in the total volume of HO lesions as assessed by CT using AHO (Baseline to week 28)
[3] TWA (standardized AUC) of the percent change in TLA by ^{18}F-NaF PET using AHOC (baseline to week 28)
[4] Percent change in the total volume of HO lesions as assessed by CT using AHOC (baseline to week 28)
Key secondary outcome measures
[5] TWA (standardized AUC) of the change in daily pain due to FOP, as measured using the daily NRS using AHO (baseline to week 28)
[6] TWA (standardized AUC) of the change in daily pain due to FOP, as measured using the daily NRS using AHOC (baseline to week 28)
Other secondary outcome measures
Percent change in ^{18}F-NaF SUVmax of individual active HO site(s) by PET using AHOC (baseline to week 8)
Percent change in ^{18}F-NaF SUVmax of individual active HO site(s) by PET using AHO (baseline to week 8)
Change in number of HO lesions as assessed by ^{18}F-NaF PET using AHOC (baseline to week 28)
Change in number of HO lesions as assessed by ^{18}F-NaF PET using AHO (baseline to week 28)
Change in number of HO lesions as assessed by ^{18}F-NaF PET using FAS (baseline to week 28)
Change in number of HO lesions detectable by CT using AHOC (baseline to week 28)
Change in number of HO lesions detectable by CT using AHO (baseline to week 28)
Change in number of HO lesions detectable by CT using FAS (baseline to week 28)

TWA (standardized AUC) of the change from baseline in daily pain due to FOP, as measured using the daily NRS using FAS (baseline to week 28)
TWA (standardized AUC) of the percent change from baseline in biomarkers of bone formation levels (including total procollagen type 1 N-Terminal propeptide, bone-specific alkaline phosphatase, and total alkaline phosphatase) in serum over 28 weeks using FAS (up to week 28)
Exploratory outcome measures
Change from baseline in CAJIS total score to week 28 using AHO (baseline to week 28)
Change from baseline in EQ-5D-3L total score to week 28 using AHO (baseline to week 28)
Patient-reported assessment of activities of daily living by FOP I-ADL (baseline to week 28)
Number and duration of flare-ups by patient e-diary (defined as experiencing ≥2 of the following: new onset of pain, swelling, joint stiffness, decrease in movement, or detection of HO) (baseline to week 28)
Location and severity of FOP disease signs and symptoms by patient e-diary including 1) pain, 2) swelling, 3) joint stiffness, 4) decreased movement, each scored using a four-point scale (0: no symptom; 1: mild; 2: moderate; 3: severe) (baseline to week 28)
Total dosage of glucocorticoid use over time
Number of patients with new HO lesions as assessed by ^{18}F-NaF PET using AHO (baseline to week 28)
Change in number of lesions that are only ^{18}F-NaF PET detectable at baseline to CT detectable lesions at week 28 (baseline to week 28)
Change from baseline in the FEV1 of spirometry at week 28 (baseline to week 28)
TWA (standardized AUC) change from baseline in the levels of hs-CRP (baseline to week 28)
**Period 2[b]**
Primary safety endpoint
Incidence and severity of TEAEs through the end of the Period 2 (baseline to week 56)
Primary outcome measures
[1] Number of new HO lesions as assessed by CT at week 56 relative to week 28 using AHO (in patients switching from placebo to garetosmab after Period 1) (week 28 to week 56)
Key secondary outcome measures
[2] Total volume of new HO lesions in patients switching from placebo to garetosmab after Period 1 as assessed by CT using AHO (week 28 to week 56)
[3] Number of new HO lesions as assessed by PET at week 56 relative to week 28 using AHO (in patients switching from placebo to garetosmab after Period 1) (week 28 to week 56)

[4] TLA by ^{18}F-NaF PET in new HO lesions in patients switching from placebo to garetosmab after Period 1 using AHO (week 28 to week 56)
[5] Percentage of patients switching from placebo to garetosmab after Period 1 with new HO lesions as assessed by CT using AHO (week 28 to week 56)
[6] Percentage of patients switching from placebo to garetosmab after Period 1 with new HO lesions as assessed by ^{18}F-NaF PET using AHO (week 28 to week 56)
Other secondary outcome measures
Number of new HO lesions in patients switching from placebo to garetosmab after Period 1 as assessed by CT using AHO (week 28 to week 56)
Total volume of new HO lesions as assessed by CT in patients switching from placebo to garetosmab after Period 1 using AHO (week 28 to week 56)
Percent of patients with new HO lesions as assessed by CT in patients switching from placebo to garetosmab after Period 1 using AHO (week 28 to week 56)
Percentage of patients switching from placebo to garetosmab after Period 1 with investigator-assessed flare-ups using AHO (week 28 to week 56)
Percentage of patients switching from placebo to garetosmab after Period 1 with flare-ups assessed by patient e-diary using AHO (week 28 to week 56)
Number of new HO lesions in patients who continue garetosmab after Period 1 as assessed by CT using AHO (baseline to week 56)
Number of new HO lesions as assessed by CT in patients who continue garetosmab after Period 1 using AHO (baseline to week 56)
Number of new HO lesions as assessed by CT in patients who continue garetosmab after Period 1 using AHO (week 28 to week 56)
Total volume in new HO lesions as assessed by CT in patients who continue garetosmab after Period 1 using AHO (baseline to week 56)
TLA in new HO lesions as assessed by ^{18}F-NaF PET in patients who continue garetosmab after Period 1 using AHO (baseline to week 56)
Percent of patients with new HO lesions as assessed by ^{18}F-NaF PET in patients who continue garetosmab after Period 1 using AHO (baseline to week 56)
Percent change from week 28 in SUV_{max} to week 56 in patients switching from placebo to garetosmab after Period 1 using AHO (week 28 to week 56)
Percent change from baseline in SUV_{max} to week 56 in patients who continue garetosmab after Period 1 using AHO (baseline to week 56)
Percent change from baseline in TLA by ^{18}F-NaF PET using AHO (baseline to week 56)

Percent change in the total volume of HO lesions in patients switching from placebo to garetosmab after Period 1 versus the same patients between baseline and week 28 as assessed by CT using AHO (baseline to week 56)
Percent change from baseline in the total volume of HO lesions as assessed by CT using AHO (baseline to week 56)
Change in number of HO lesions in patients switching from placebo to garetosmab after Period 1 as assessed by ^{18}F-NaF PET from week 28 to week 56 versus the same patients between baseline and week 28 using AHO (baseline to week 28; week 28 to week 56)
Change in number of HO lesions in patients switching from placebo to garetosmab after Period 1 as assessed by CT scan from week 28 to week 56 versus the same patients between baseline and week 28 using AHO (baseline to week 28; week 28 to week 56)
Exploratory outcome measures
Change in the total volume of new HO lesions as assessed by CT in patients switching from placebo to garetosmab after Period 1 using AHO (week 28 to week 56)
Joint function assessment by physician at week 56 by the CAJIS using AHO (week 28 to week 56)
Patient-reported quality of life by EQ-5D-3L at week 56 using AHO (week 28 to week 56)
Patient-reported assessment of activities of daily living by FOP I-ADL at week 56 using AHO (week 28 to week 56)
Number and duration of flare-ups by patient e-diary (defined as experiencing ≥2 of the following: new onset of pain, swelling, joint stiffness, decrease in movement, or detection of HO) (week 28 to week 56)
Location and severity of FOP disease signs and symptoms by patient e-diary including 1) pain, 2) swelling, 3) joint stiffness, 4) decreased movement, each scored using a 4-point scale (0: no symptom; 1: mild; 2: moderate; 3: severe) (week 28 to week 56)
Number of new HO lesions as assessed by ^{18}F-NaF PET in patients who continue garetosmab after Period 1 using AHO (baseline to week 56)
Change in number of HO lesions by CT using AHO (baseline to week 56)
Number of patients with new HO lesions as assessed by ^{18}F-NaF PET using AHO (baseline to week 56)
Percent change from baseline of mean SUV_{mean} of selected normal bones (e.g., lumbar spine and femoral heads) as assessed by ^{18}F-NaF PET/CT using AHO (baseline to week 56)
Percent change from baseline in venous plasma clearance ^{18}F-NaF SUV using AHO (baseline to week 56)
Percent change from baseline in ^{18}F-NaF incorporation rate (Ki) in individual active HO lesion(s) using AHO (baseline to week 56)

Percent change from baseline of ratio of SUV_{max} of individual active HO lesion(s) to venous plasma SUV during PET scan using AHO (baseline to week 56)
Change from baseline in the FEV1 of spirometry using AHO (baseline to week 56)
Change from baseline in the FEV1 of spirometry using AHO (baseline to week 28)

Endpoints were ordered per protocol. Period 2 endpoints- new hypothesis were refined based on Period 1

[a]Testing of the of the primary and key secondary followed a hierarchical testing procedure to address multiplicity at an overall two-sided alpha of 0.05 significance for Period 1 and order is denoted in the table.

[b]To control the type-I error rate at 0.10 for the primary and key secondary endpoints, a hierarchical testing procedure was applied at a two-sided significance level for Period 2.

^{18}F-NaF, fluorine-18-labelled sodium fluoride; ADA, anti-drug antibody; AHO, active heterotopic ossification analysis set; AHOC, active heterotopic ossification classic mutation analysis set; AUC, area under the curve; CAJIS, Cumulative Analog Joint Involvement Scale; CT, computed tomography; EQ-5D-3L, EuroQol five dimensions questionnaire with a three-level scale; FAS, full analysis set; FEV, forced expiratory volume in 1 second; FOP, fibrodysplasia ossificans progressiva; HO, heterotopic ossification; NRS, numeric rating scale; PET, positron emission tomography; PK, pharmacokinetic; SUV_{max}, maximum standardized uptake value; SUV_{mean}, mean standardized uptake value; TEAE, treatment-emergent adverse event; TLA, total lesion activity; TWA, time-weighted average.

Figure S1. Activin A activates signaling by FOP-mutant ACVR1 but inhibits signaling by wild type ACVR1

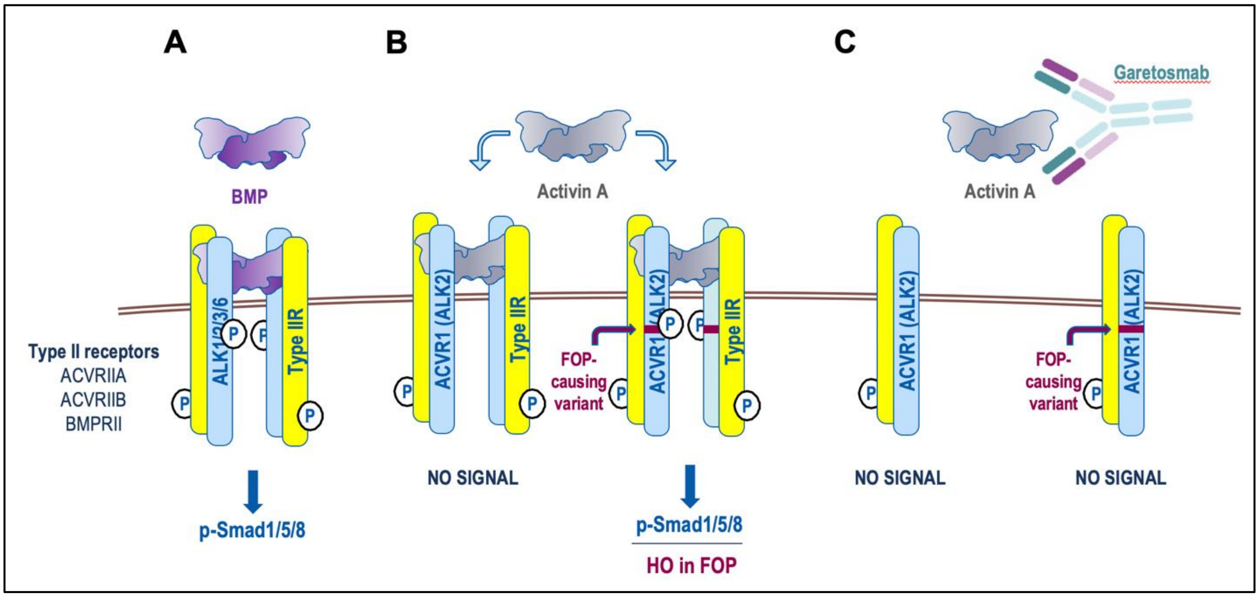

(**A**) In the presence of BMPs, ACVR1 forms a signaling complex together with its cognate type II receptors, ACVR2A, ACVR2B, and BMPR2. This complex activates signaling, mainly via phosphorylation of Smad1/5/8. (**B**) In the presence of activin A, wild type ACVR1 forms a non-signaling complex that antagonizes signaling by BMPs. When ACVR1 is an FOP-causing variant, the non-signaling complex is converted into a signaling complex and transduces signal much like the complex formed by BMPs. (**C**) Garetosmab blocks the interaction of activin A with its cognate type I receptors ACVR1 (ALK2) and ACVR1B (ALK4, not depicted), and hence inhibits the formation of both signaling and non-signaling complexes that would normally be generated by activin A and the corresponding type I and type II receptor pairs.

BMP, bone morphogenetic protein; FOP, fibrodysplasia ossificans progressiva; HO, heterotopic ossification; R, receptor.

Figure S2. Schematic overview of the study design

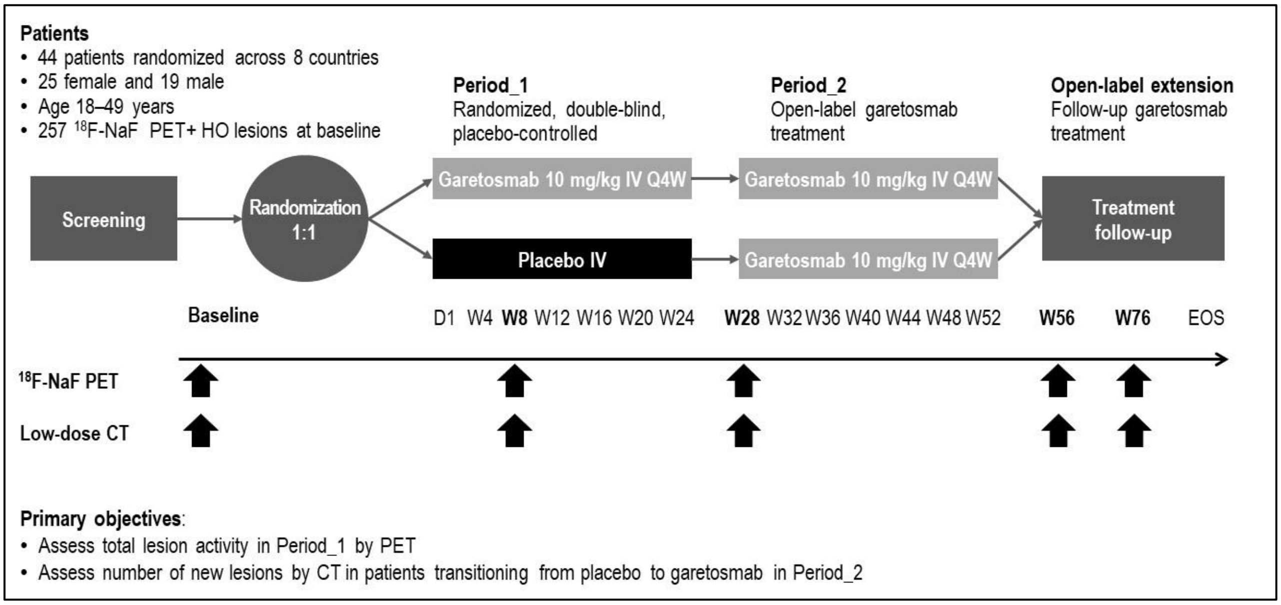

LUMINA-1 is a phase 2, randomized, double-blind, placebo-controlled study in adult patients with FOP. This study consists of a 4-week screening/baseline period, a 28-week randomized double-blind placebo-controlled treatment period (Period 1), a 28-week open-label garetosmab treatment period (Period 2), and a follow-up treatment period with open-label garetosmab (open-label extension). Following confirmed eligibility during the screening/baseline period (day –28 to day 1), patients were randomized 1:1 to 10 mg/kg garetosmab IV dosed Q4W or placebo. Efficacy is assessed by ^{18}F-NaF PET and low-dose CT imaging analysis of HO at weeks 8, 28, 56, and 76. The primary analysis was conducted when all the patients completed the double-blind treatment (Period 1). Actual enrollment matches the prespecified enrollment as per protocol.

^{18}F-NaF, fluorine-18-labelled sodium fluoride; CT, computed tomography; D, day; EOS, end of study; FOP, fibrodysplasia ossificans progressiva; HO, heterotopic ossification; IV, intravenous; PET, positron emission tomography; Q4W, every 4 weeks; W, week.

Figure S3. BMP9 levels in patients who experienced TEAEs of epistaxis over time

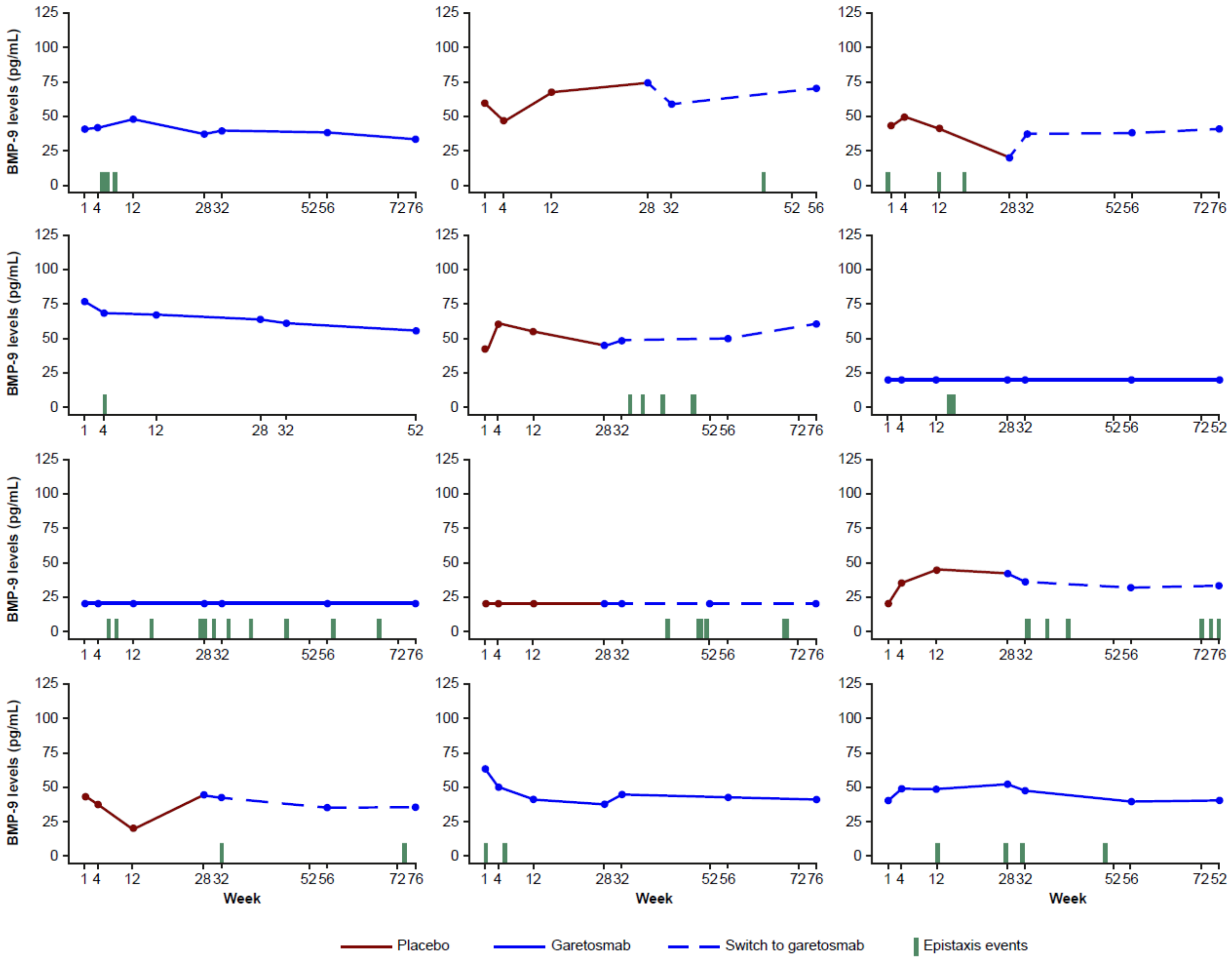

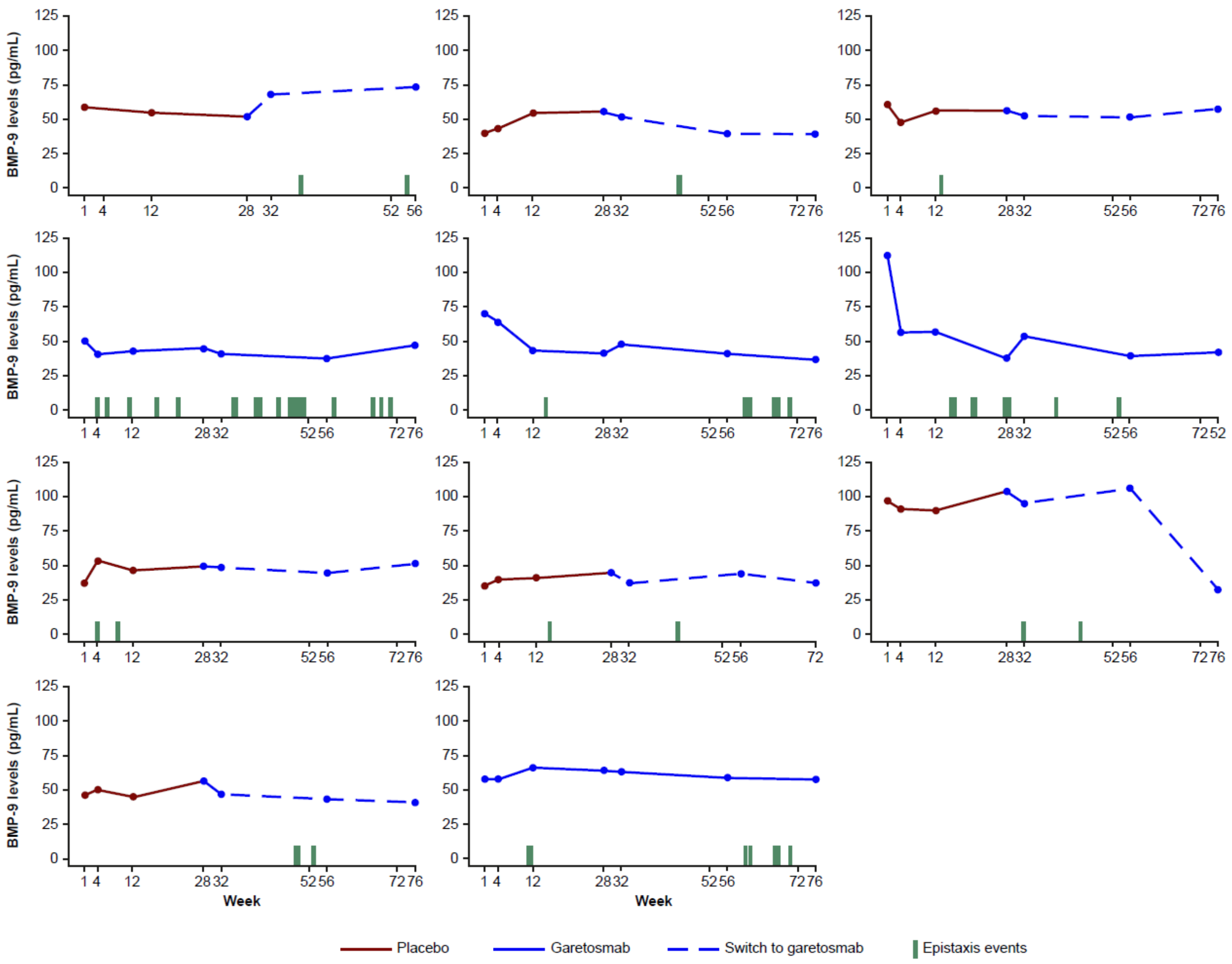

Red lines indicate an epistaxis event.

BL, baseline; BMP9, bone morphogenetic protein 9; TEAEs, treatment-emergent adverse events.
