# Supplementary material for: Garetosmab in fibrodysplasia ossificans progressiva: a randomized, double-blind, placebo-controlled phase 2 trial
Source: Nat Med. 2023 Sep 28;29(10):2615–24. doi: 10.1038/s41591-023-02561-8 (PMC10579054; doi:10.1038/s41591-023-02561-8)
Supplement: Supplementary file 2 — Reporting Summary [file 41591_2023_2561_MOESM2_ESM.pdf]

nature portfolio

Corresponding author(s): Scott Mellis

Last updated by author(s): Aug 21, 2023

Reporting Summary

Nature Portfolio wishes to improve the reproducibility of the work that we publish. This form provides structure for consistency and transparency in reporting. For further information on Nature Portfolio policies, see our Editorial Policies and the Editorial Policy Checklist.

Statistics

For all statistical analyses, confirm that the following items are present in the figure legend, table legend, main text, or Methods section.

n/a	Confirmed	
☐	☒	The exact sample size (*n*) for each experimental group/condition, given as a discrete number and unit of measurement
☐	☒	A statement on whether measurements were taken from distinct samples or whether the same sample was measured repeatedly
☐	☒	The statistical test(s) used AND whether they are one- or two-sided<br>*Only common tests should be described solely by name; describe more complex techniques in the Methods section.*
☐	☒	A description of all covariates tested
☒	☐	A description of any assumptions or corrections, such as tests of normality and adjustment for multiple comparisons
☐	☒	A full description of the statistical parameters including central tendency (e.g. means) or other basic estimates (e.g. regression coefficient) AND variation (e.g. standard deviation) or associated estimates of uncertainty (e.g. confidence intervals)
☐	☒	For null hypothesis testing, the test statistic (e.g. *F*, *t*, *r*) with confidence intervals, effect sizes, degrees of freedom and *P* value noted<br>*Give P values as exact values whenever suitable.*
☒	☐	For Bayesian analysis, information on the choice of priors and Markov chain Monte Carlo settings
☒	☐	For hierarchical and complex designs, identification of the appropriate level for tests and full reporting of outcomes
☒	☐	Estimates of effect sizes (e.g. Cohen's *d*, Pearson's *r*), indicating how they were calculated

Our web collection on statistics for biologists contains articles on many of the points above.

Software and code

Policy information about availability of computer code

Data collection	Clinical data were captured in the clinical database using the Medidata Rave Electronic Capture Data system.
Data analysis	Clinical data were analyzed using SAS 9.4.

For manuscripts utilizing custom algorithms or software that are central to the research but not yet described in published literature, software must be made available to editors and reviewers. We strongly encourage code deposition in a community repository (e.g. GitHub). See the Nature Portfolio guidelines for submitting code & software for further information.

Data

Policy information about availability of data

All manuscripts must include a data availability statement. This statement should provide the following information, where applicable:

- Accession codes, unique identifiers, or web links for publicly available datasets
- A description of any restrictions on data availability
- For clinical datasets or third party data, please ensure that the statement adheres to our policy

Qualified researchers may request access to study documents that support the methods and findings reported in this manuscript. Individual anonymized patient data will be considered for sharing once the product and indication has been approved by major health authorities (eg, FDA, EMA, PMDA, etc), if there is legal authority to share the data and there is not a reasonable likelihood of patient re-identification. Submit requests to https://vivli.org/.

Human research participants

Policy information about studies involving human research participants and Sex and Gender in Research.

Reporting on sex and gender	Sex and/or gender was not considered in the study design. The sex of participants was self-reported, and was summarized as part of the baseline characteristic information collected for the trial. The trial enrolled 44 patients with FOP; 25 (56.8%) were female and 19 (43.2%) were male.
Population characteristics	The study population consisted of male and female patients aged 18–60 years with a clinical diagnosis of FOP and a history of FOP disease activity within 1 year of screening, and documentation of ACVR1 mutation. In addition, patients had to be willing and able to attend and comply with study visits and to undergo PET and CT imaging procedures.
Recruitment	Male and female patients aged 18–60 years with a clinical diagnosis of FOP and a history of FOP disease activity within 1 year of screening, and documentation of ACVR1 mutation were recruited. Patients were excluded if they used bisphosphonate therapies within 1 year of screening, as these medications alter bone metabolism and would confound the primary efficacy analysis. To address a potential risk of embryotoxicity or male reproductive organ toxicity, the protocol excluded pregnant or breastfeeding women, as well as women of child-bearing potential and men who were unwilling to practice highly effective contraception.
Ethics oversight	Patient safety and welfare were monitored by an Independent Data Monitoring Committee. This study was conducted in accordance with the 2013 Declaration of Helsinki the International Council for Harmonization guidelines for Good Clinical Practice, and SAGER guidelines. All patients provided written, informed consent.<br>LUMINA-1 (NCT03188666) was conducted at 11 sites in eight countries. The full protocol is available online. The trial was approved by the following institutional review boards: University Health Network 700 University Ave.10th Floor, Suite 1056, Toronto Ontario, M5G1Z5, Canada; Comité de Protection des Personnes (CPP) Ile-de-F, 78 rue du Général Leclerc, Le Kremlin Bicentre, Paris France, 94275; Comitato Etico Regione Liguria, IRCCS Ospedale Policlinico, San Martino, Largo Rosanna Benzi, 10, Genova, Italy, 16132; Science Committee AMS, VUmc, Internal Medicine, Room 4A35, De Boelelaan 1117, 1081 HV Amsterdam, The Netherlands; METC VUmc BS7, Kamer H-443, Postbus 7057, Amsterdam, The Netherlands, 1007 MD; Komisja Bioetyczna Uniwersytetu Rzeszowskiego ul. Warszawska 26A, 35-205, Rzeszow, Poland; Comité de Ética de la Investigación con medicamentos del Hospital Universitario Ramón y Cajal. Ctra. Colmenar, km. 9,100, Madrid, Spain, 28034; London - Central Research Ethics Committee, 3rd Floor, Barlow House, 4 Minshull Street, Manchester, UK, M1 3DZ; University of Pennsylvania, Office of Regulatory Services, 3624 Market Street, Suite 301 S, Philadelphia, 19104, USA; Mayo Clinic Institutional Review Board, 200 First Street SW, Rochester, Minnesota, 55905, USA; Vanderbilt University 1313 21st Ave., South, Suite 505, Nashville, Tennessee, 37232, USA.

Note that full information on the approval of the study protocol must also be provided in the manuscript.

Field-specific reporting

Please select the one below that is the best fit for your research. If you are not sure, read the appropriate sections before making your selection.

☒ Life sciences ☐ Behavioural & social sciences ☐ Ecological, evolutionary & environmental sciences

For a reference copy of the document with all sections, see nature.com/documents/nr-reporting-summary-flat.pdf

Life sciences study design

All studies must disclose on these points even when the disclosure is negative.

Sample size	The sample size estimation for at least 24 patients (12 patients per treatment group) with active HO at baseline and classic ACVR1[R206H] mutation is based on statistical considerations for the following efficacy endpoints: percent change from baseline in (1) total lesion activity by 18F NaF PET over 28 weeks, (2) total volume of HO lesion by CT at week 28, and (3) 18F-NaF SUVmax at week 8. Accounting for a 20% dropout rate at week 28, the sample size would yield approximately 10 patients per treatment group for week-28 analyses. This sample size will provide 80% power at a two-sided 0.05 significance level in allowing the detection of an observed treatment difference in the order of 57%, 65%, and 40% reduction in the total lesion activity by 18F-NaF PET, the total volume of HO lesion by CT, and the 18F-NaF SUVmax, respectively, based on other bone diseases, including FOP30 and modeling in FOP mice.17,18,33<br>In Period 1, testing of the primary and key secondary efficacy endpoints followed a hierarchical testing procedure to address multiplicity at an overall two-sided alpha = 0.05 significance level. Testing of the key secondary efficacy endpoints will follow a hierarchical testing sequence only if statistical significance is established for all primary endpoints. No further adjustments was made for other secondary and exploratory endpoints, for which nominal P-values will be provided for descriptive purpose only. Safety outcomes were analyzed using descriptive statistics.<br>In Period 2, the evaluation of these prospectively specified re-defined primary and key secondary endpoints related to new heterotopic bone formation warranted new analyses, which were independent from that of Period 1 and required their own overall type I error rate of 10% (alpha of 0.1). To control the type-I error rate at 0.10 for the primary and key secondary null hypotheses in Period 2, a hierarchical testing procedure was applied at a two-sided 10% significant level as detailed. No further adjustments were made for other secondary and exploratory endpoints in Period 2, for which estimates, 95% CI, and/or nominal P-values will be provided for descriptive purpose. Safety outcomes were analyzed using descriptive statistics.
Data exclusions	No data were excluded from the analyses

Replication	Not applicable for clinical data
Randomization	Enrolled patients were randomized (1:1) to receive garetosmab 10 mg/kg Q4W, as previously assessed,26 or placebo in Period 1, according to a central randomization scheme. Block randomization was done using an interactive response technology provided to the designated study pharmacist or qualified designee. Randomization was stratified by presence/absence of baseline active HO lesions, sex, and mutation type. All PET/CT scans were reviewed by two independent readers and an adjudicator; all three were blinded to treatment assignment.
Blinding	Blinding procedures are described in detail in the methods section of the manuscript. Regarding imaging, all PET/CT scans were reviewed by two independent readers and an adjudicator; all three were blinded to treatment assignment.

Reporting for specific materials, systems and methods

We require information from authors about some types of materials, experimental systems and methods used in many studies. Here, indicate whether each material, system or method listed is relevant to your study. If you are not sure if a list item applies to your research, read the appropriate section before selecting a response.

Materials & experimental systems

n/a	Involved in the study	
☐	☒	Antibodies
☒	☐	Eukaryotic cell lines
☒	☐	Palaeontology and archaeology
☒	☐	Animals and other organisms
☐	☒	Clinical data
☒	☐	Dual use research of concern

Methods

n/a	Involved in the study	
☒	☐	ChIP-seq
☒	☐	Flow cytometry
☒	☐	MRI-based neuroimaging

Antibodies

Antibodies used

Kit reagents were used as described: Recombinant human BMP-9 Standard (R&D Systems, Catalog/Part # 843430); Mouse anti-human BMP-9 monoclonal antibody (R&D Systems, Catalog/Part # 843428) (This is the Capture Antibody, which is used at the working conc. of 2 µg/mL); Biotinylated goat anti-human BMP-9 polyclonal antibody (R&D Systems, Catalog/Part # 843429) (This is the Detection Antibody, which is used at the working conc. of 50 ng/mL)

Mouse anti-human BMP-9 monoclonal antibody produced by Regeneron Pharmaceuticals, Inc. at Rensselaer, NY facility
biotinylated goat anti-human BMP-9 polyclonal antibody produced by Regeneron Pharmaceuticals, Inc. at Rensselaer, NY facility

Validation

Manufacturing site information is as follows:
Regeneron Pharmaceuticals, Inc.
81 Columbia Turnpike
Rensselaer, NY 12144
USA

Date of Manufacture (DOM) and Lot ID #s supplied to clinic are as follows:
DOM Lot ID
21-Jan-16 8171800005
21-Jan-16 8171800006
8-Dec-16 8199800001
8-Dec-16 8199800002
8-Dec-16 8199800007
8-Dec-16 8199800002
8-Dec-16 8199800002
5-Feb-18 8199800010
5-Feb-18 8199800014
5-Feb-18 8199800020
21-Jul-19 8275300001
6-Oct-19 8275300003
6-Oct-19 8275300004
1-Apr-20 8275300010
28-Aug-20 8275300006

Clinical data

Policy information about clinical studies
All manuscripts should comply with the ICMJE guidelines for publication of clinical research and a completed CONSORT checklist must be included with all submissions.

Clinical trial registration: NCT03188666

Study protocol

The full protocol is available online.

Data collection

The study was initiated in February 2018; primary data cut-off was September 17, 2019 (week 28); additional data cut-off dates for efficacy analyses were August 11, 2020 (week 56), and October 30, 2020 (week 76); safety was reported until last patient last visit on September 16, 2021. The study was conducted at 11 sites in eight countries across North America and Europe.

Outcomes

Period 1: The pre-specified primary endpoint for efficacy was the effect of garetosmab vs. placebo on time-weighted average (TWA) of the percentage change from baseline in TLA by PET; TLA is considered proportional to the deposition rate of bone mineral into actively forming HO lesions. The next endpoint in the hierarchy was to assess the percentage change in the total volume of HO lesions by CT in Period 1 relative to baseline. The last endpoint in the hierarchy was TWA change from baseline in daily pain due to FOP as measured using the daily numeric rating scale (NRS) over 28 weeks in AHO and AHOC. A full list of secondary and exploratory endpoints is provided in Table S14. Exploratory endpoints in Period 1 included the percentage of patients with flare-ups as assessed by patient diary and post-hoc analyses included investigator reported flare-ups.
Period 2: Based on the outcomes of Period 1, the pre-specified primary endpoint for efficacy for Period 2 was prospectively changed to the number of new lesions in patients crossing-over from placebo to garetosmab as assessed by CT. Additionally, we assessed efficacy at week 56 relative to week 28 in total volume of new HO lesions by CT, the number of new lesions by PET, total lesion activity by PET in new HO lesions, and percent of patients with new lesions by CT and PET. A full list of secondary and exploratory endpoints is provided in Table S14.
Safety: The primary safety endpoint for Period 1 was the incidence and severity of AEs, which included both those not present at baseline and those which were an exacerbation of a pre-existing condition. A full safety profile to end of study was descriptively reported.

No sex- or gender-based analyses have been performed as these were not pre-specified in the study protocol/statistical analysis plan for this trial. Post-hoc sex- or gender-based analyses have not been performed due to the small sample sizes within treatment groups.
